# Assessing Acetyl-Coenzyme A Carboxylase Activity and Inhibition in *Caenorhabtidis elegans* Using a Partially Purified Protein Extract

**DOI:** 10.1021/acs.analchem.5c00828

**Published:** 2025-08-26

**Authors:** Gennaro Battaglia, Yamanappa Hunashal, Suma Gopinadhan, Hin Hark Gan, Yasmine Moussa, Fathima S. Mohammed Refai, Angela Amoresano, Hala Zahreddine Fahs, Kristin C. Gunsalus, Gennaro Esposito, Fabio Piano

**Affiliations:** † Science Division, 167632 New York University Abu Dhabi, Saadiyat Island, Abu Dhabi, United Arab Emirates; ‡ Dipartimento di Scienze Chimiche, Università di Napoli “Federico II”, 80138 Naples, Italy; § Center for Genomics and Systems Biology, New York University Abu Dhabi, Saadiyat Island, Abu Dhabi, United Arab Emirates; ∥ Center for Genomics and Systems Biology, Department of Biology, New York University, New York, New York 10003, United States; ⊥ Dipartimento di Medicina, Università di Udine, 33100 Udine, Italy; # Istituto Nazionale Biostrutture e Biosistemi, 00136 Rome, Italy

## Abstract

Acetyl-coenzyme A carboxylase (ACC) catalyzes the ATP-dependent carboxylation of acetyl-CoA into malonyl-CoA, the first step in fatty acid biosynthesis. ACC is increasingly recognized as being crucial for energy metabolism, leading to its emergence as a potential therapeutic target mainly for obesity and cancer. All previous ACC kinetics studies were conducted with pure enzyme preparations and measurements at several substrate concentrations using methods such as radioactivity counting of labeled substrate(s) and/or product(s) or UV estimation of chromatographically resolved components. In alternative, a real-time kinetics method based on HPLC or NMR monitoring was developed and successfully applied using partially purified protein extracts and a single substrate concentration. Kinetic parameters were derived for *C. elegans* ACC in the absence and presence of the ACC inhibitor avocadene acetate, a compound present in avocadoes. Both monitoring techniques yielded consistent kinetic parameters that compared well with previously reported values and provided new insights into the inhibition mechanism.

## Graphical abstract

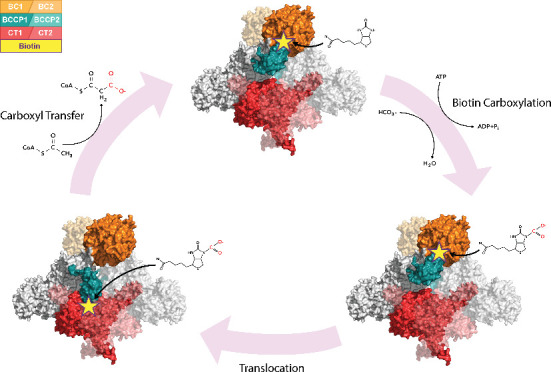

## Introduction

Acetyl-CoA carboxylase (ACC) is a crucial enzyme that catalyzes the rate-limiting step in *de novo* fatty acid biosynthesis, producing malonyl-CoA.^1^ This metabolite is essential for both the synthesis and breakdown of fatty acids,^1,2^ providing a two-carbon building block and the energy required for elongating fatty acids of various chain lengths. Fatty acids are vital for energy storage, membrane formation, and lipid signaling. In eukaryotes, malonyl-CoA also inhibits fatty acid degradation by blocking the transport of palmitoyl-CoA into mitochondria, thereby regulating mitochondrial β-oxidation, which impacts ATP production.^1–4^ The concentration of malonyl-CoA is therefore critical for maintaining metabolic balance, making ACC a strategic target for therapeutic interventions in disorders related to fatty acid metabolism, such as obesity, cancer, and hepatic steatosis.^5–9^

ACC is evolutionarily conserved in various organisms. In prokaryotes and plant plastids, it consists of multiple (3–4) subunits,^1,10,11^ while in eukaryotes, these subunits have fused into a single multidomain protein that operates as a homodimer, capable of forming filamentous structures.^3,7–13^ Whereas invertebrates possess a single ACC enzyme,^14,15^ in mammals, ACC activity is partitioned between two paralogous enzymes: ACC1, primarily found in liver and adipose tissues, and ACC2, associated with the mitochondrial outer membrane and enriched in muscle.^2,12,16,17^ This distribution indicates that ACC1 is mainly involved in lipogenesis, whereas ACC2 regulates lipolysis.

The enzymatic reaction that converts acetyl-CoA to malonyl-CoA involves two steps (Figure 1). First, a biotin cofactor undergoes carboxylation in the biotin carboxylase (BC) domain of one ACC subunit. Second, the carboxyl group is transferred to acetyl-CoA in the carboxyl transferase (CT) domain of the other subunit.^3,12–14,18,19^ This first step is powered by ATP hydrolysis, which also supports the second exergonic step.

**Figure 1:**
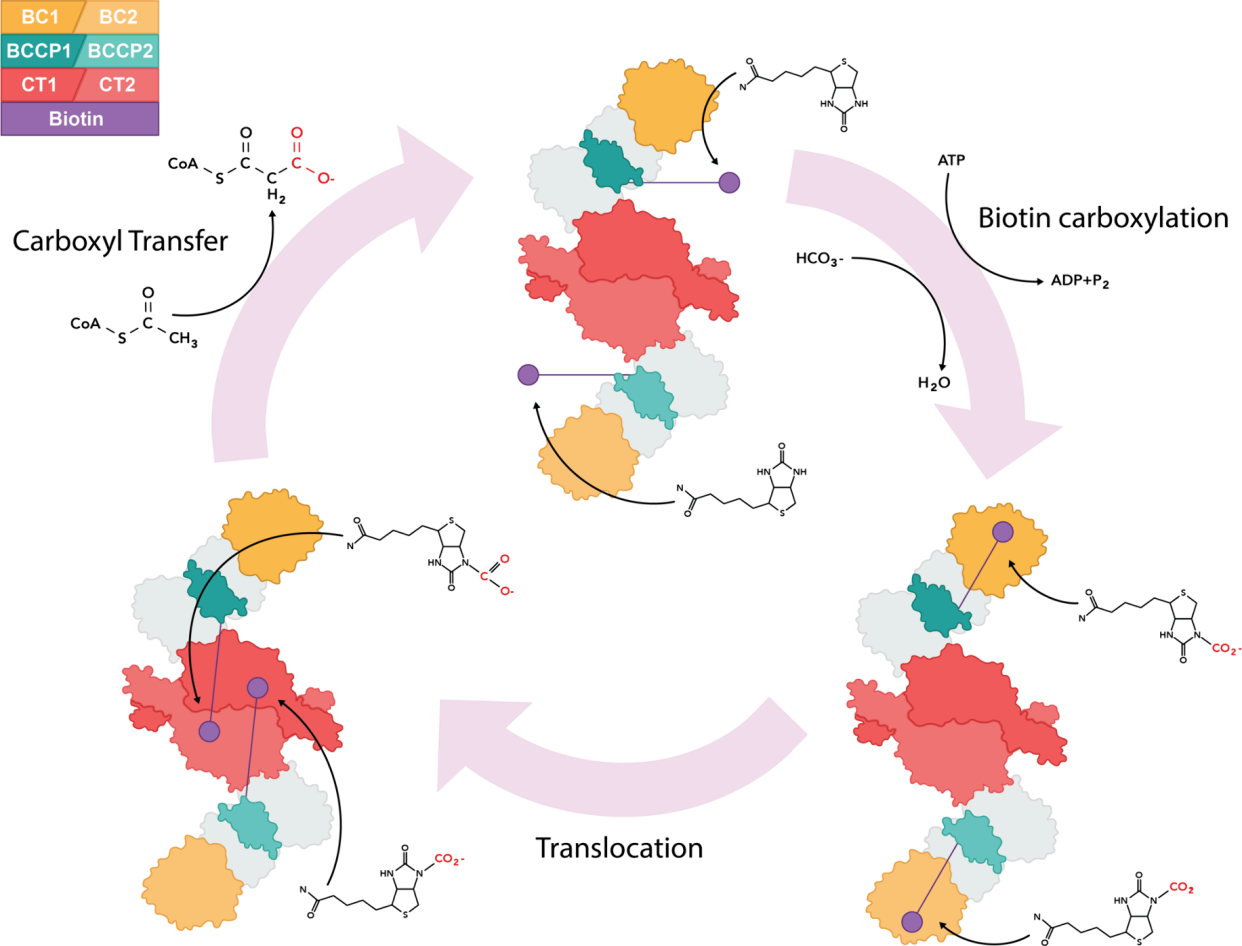
ACC enzyme converts acetyl-CoA to malonyl-CoA in a two-stage reaction cycle, requiring both subunits of a homodimer. Two ACC monomers are activated by homodimerization. Different color intensities indicate the homodimer subunit of key domains BC (ochre), CT (red) and BCCP (green), according to the upper-left code. Biotin (purple circle) is carboxylated in the BC domain of each subunit, using bicarbonate from dissolved CO_2_, and energy from ATP hydrolysis. Carboxybiotin translocates to the active site in the CT domain of the other subunit of the homodimer. The carboxyl group is transferred from the biotin carbamate to the enolate of the acetyl moiety of acetyl-CoA, producing malonyl-CoA. Only the relevant ACC domains are labeled. Two additional domains with no catalytic role, BT (BC-CT interaction) and CD (central domain), are shown in the illustration in pale gray.^3,13,14,18^

In the model organism *Caenorhabditis elegans* (*C. elegans*), the gene *pod-2* encodes a typical ACC, also known as POD-2.^20–24^ ACC activity is essential across all developmental stages, and disruption of *pod-2* function during oogenesis leads to loss of eggshell integrity, significant embryonic defects, and embryonic lethality.^20,25,26^

Chemical inhibition of ACC has potential therapeutic applications against parasitic nematodes, which pose serious risks for animal health and economic challenges in agriculture.^27–29^ For example, the insecticide spirotetramat disrupts development and causes lipid depletion in *C. elegans* and other nematodes.^30^ Recently, we identified a family of avocado-derived fatty alcohols and acetates (AFAs) that inhibit *C. elegans* ACC and cause developmental arrest and lethality at all life stages.^31^ The rat liver ACC inhibition by some AFA compounds was earlier reported,^32^ but our study also showed that these compounds inhibit various parasitic nematodes *in vitro* and *in vivo* without harming mammalian hosts.^31^

To further characterize AFA inhibition of *C. elegans* ACC, we have developed quantitative *in vitro* approaches that exploit HPLC (high-performance liquid chromatography) and NMR (nuclear magnetic resonance) monitoring. The methodology was applied using a partially purified protein extract without and with avocadene acetate (the acetate derivative of a 17-carbon AFA). Whereas most previous measurements of ACC activity have been carried out with the classical method of initial rate determinations at several substrate concentrations using radiolabeled substrates—either with pure ACC preparations or with crude or fractionated protein extracts^30,33–37^ —we demonstrate that our approach achieves robust measurement of relative activity and chemical inhibition of ACC present in a partially purified extract. By obviating the need for expensive radioactive labeling of substrates, laborious and time-consuming protein purification protocols, and demanding series of measurements at different substrate concentrations, the approach described here represents a convenient alternative to previous methods to assess ACC activity.

## Experimental Section

### Chemicals and Reagents

See the Supporting Information (SI).

#### ACC preparation

The *C. elegans* strain PHX1772 *pod-2­(syb1772­[pod-2::His10])* expressing (His)_10_-tagged ACC was used for the ACC pull-down procedure. The protein extract was prepared from 500 000 fourth-stage larvae (L4), grown in liquid culture. The worms were pelleted, washed three times with M9 buffer, and resuspended in the lysis buffer (50 mM HEPES, 150 mM NaCl, 1 mM DTT or TCEP, 10% glycerol, 1% protease inhibitor cocktail Halt). The lysed suspension was then added dropwise to a liquid N_2_ precooled mortar. The frozen beads were ground to a fine powder. This powder was thawed again and homogenized by using a QSonica sonicator. Three mild sonication cycles were applied (15 s power on, 15 s power off) interleaved by 3 min of cooling on ice. The sample was then centrifuged at 50 000 rpm in a Beckman Coulter Optima XPN-90 Ultracentrifuge using Type 90 Ti rotor at 4 °C. The clear supernatant was then transferred to equilibrated Ni-NTA agarose resin slurry column, and the mix was incubated for 1 h at 4 °C. After collecting the flow through, the column was washed five times with the wash buffer (50 mM HEPES, 150 mM NaCl, 20 mM imidazole, 1 mM DTT or TCEP, 10% glycerol) and the immobilized protein was eluted with 250 mM imidazole buffer. The eluted fraction was then concentrated by ultrafiltration (100 kDa cutoff), and the collected fraction was used for the chromatographic and spectroscopic assays.

### HPLC Kinetics

The ACC kinetic assay was carried out with the HPLC-DAD system (see the SI), using two eluent solvents: (A) 10 mM K_2_HPO_4_ and (B) CH_3_OH. The chromatographic runs were conducted with an eluent composition scheme adapted from a previous report^36^ as follows: 0–1 min, 0% B; 1–5 min, linear gradient 0–30% B; 5–11 min, constant 30% B; 11–12 min, linear gradient 30–0% B to restore the initial conditions, with solvent A complementing to 100% throughout the whole chromatographic separation run and the subsequent column re-equilibrating interval of 12 min (12–24 min). The first HPLC run (*t*_0_) was always performed with an automatic sampling of the HPLC injector withdrawn from the 500 μL batch of reaction mixture devoid of ACC. Following the *t*_0_ injection, an aliquot (10–20 μL) of concentrated *C. elegans* protein extract was manually added to the reaction batch to initiate the transformation monitored by automatic sampling from the reaction batch at the start of each chromatographic cycle. Thus, the collection timing between any consecutive data points was always 24 min. Acetyl-CoA, malonyl-CoA, and ATP were simultaneously detected following the absorption at 395 nm. The identification of the target species was preliminarily established using individual compound standard preparations. The typical composition of the initial reaction batches was 50 μM acetyl-CoA, 50 μM ATP, 50 mM NH_4_HCO_3_, 10 mM MgCl_2_ and, for the inhibition testing experiments, 50–100 μM avocadene acetate. A reaction mixture without ATP was also prepared to explore the relative kinetic profile. Due to insolubility in water, avocadene acetate was added as a concentrated mother solution in CH_3_CN. The relative experimental control was also performed with a sample containing only CH_3_CN without avocadene acetate. Using the relative reference standards, calibration curves for precise concentration estimates were constructed only for acetyl-CoA and malonyl-CoA, including also the corresponding determinations of the respective detection and quantification limits (SI (Table S1)). No calibration curve was determined for ATP because of the relative peak proximity to the elution front which affects the quantitative reproducibility. The ATP concentration estimate was therefore based on the rescaling of the normalized peak intensities to the initial value of 50 μM. The acetyl-CoA calibration curve was instead used for the signal attributed to free CoA based on a nearly total chromophore identity.

### NMR Analysis

The samples for NMR kinetics assessment were typically prepared by adding aliquots (μL) of acetyl-CoA and ATP mother solutions to 25 mM NH_4_HCO_3_ and 5 mM MgCl_2_ dissolved in D_2_O, to reach concentrations of ∼50 μM for both species (precisely 48.6 ± 0.8 μM and 52.8 ± 3.4 μM, respectively, for acetyl-CoA and ATP, from joint NMR and UV quantification). A sample without ATP was also prepared for the corresponding control experiment. For the assessment of inhibitory effect, a few microliters of a concentrated avocadene acetate preparation in CD_3_CN were also added to the mentioned D_2_O solutions, to reach nominal concentrations of 50 μM or 25 μM. The samples for NMR experiments were prepared using Tris-d_11_ and ethylene glycol-d_6_ to suppress overwhelming signals compromising the detection of the substrate and products peaks. Careful adjustments of the NMR acquisition conditions were preliminarily carried out on each specific substrate or substrate and inhibitor D_2_O solution, before adding an aliquot (38 μL) of ACC-containing *C. elegans* protein extract previously obtained by affinity chromatography and further treated by ultrafiltration (100 kDa threshold). The final overall protein concentration of the extract was 1.72 μg/μL, as estimated by the UV absorption at 280 nm. Typically, following the protein extract addition, the acquisitions started after a 4–5 min dead-time delay, necessary for equilibration and field homogeneity stabilization. ^1^H NMR spectra were collected at 14.0 T (^1^H resonance at 600.19 MHz) on a Bruker Avance III NMR system equipped with a triple resonance cryoprobe. All the spectra were acquired at 20 °C to reduce and desirably prevent potential stability problems of the protein extracts added to the reaction mixtures, given the longer duration of the NMR data recording, compared to HPLC. The experimental data were, in fact, collected by issuing automatic consecutive acquisition series of one-dimensional ^1^H NMR spectra, with a single series collection typically lasting 12–15 h. Each spectrum (i.e., kinetic data-point) was obtained with a relaxation delay of 1 s, a recycling time of 3.70 s, 8 dummy scans, 128 scans acquired over a sweep width of 9615 Hz and 32 768 points and required ∼6.2 min. The optimized acquisition sequence included residual HOD suppression obtained by pairing WATERGATE elements^38^ in the excitation sculpting mode.^39^ The experimental data were processed with Topspin 4.0.6 (Bruker), and the peaks of acetyl-CoA acetyl methyl, free acetate methyl, and CoA cysteamine moiety CH_2_–S of both acetyl- and malonyl-CoA of each spectrum were carefully integrated using the value of the first acquired spectrum in each series as normalization reference. The measured integrals were corrected by subtraction of the corresponding background intensity due to the contribution of the *C. elegans* protein extract. The latter contribution was evaluated from the NMR spectrum of a 38 μL extract sample, dissolved in the same D_2_O volume as the reactant samples. After recording the NMR spectrum with the same standard settings as the kinetics data-point acquisitions, the protein extract signals were carefully integrated using the same integration regions as employed to estimate the integrals of the acetyl- and malonyl-CoA thio-methylene and the acetyl-CoA acetyl methyl, i.e., the extract background overlapping signals. The extract integrals were then calibrated for each kinetic series of spectra using the first data point of the series. Subsequently, the integral values of the affected signals in all the spectra of any series were corrected by subtracting the corresponding contribution from the extract background.

### Protein Assay and Identification

Proteins were digested with trypsin following the reduction and alkylation steps. Desalted digests were analyzed by LC-MS/MS. MASCOT 2.4.0 software analyzed MS/MS data against the UniProt *C. elegans* database. See the SI for details of the procedures.

### Computational Modeling

As described previously,^31^ the POD-2 homodimer complex containing the CT active site with CoA or biotin substrate was modeled using Alphafold 2 and the solved crystal structures of yeast ACC homodimer complexes (PDB ID: 5csl, 1w2x).^14,40^ Induced-fit docking^41^ of avocadene acetate to the CT active site was performed with/without CoA using the software developed by Schrödinger LLC (2021–1 package). Avocadene acetate docking was centered at the biotin binding site, but the search volume encompassed the entire CT active site. The docking parameters were the same as reported previously.^31^

## Results

### HPLC-Based Real-Time Kinetics of ACC

Partially purified preparations of *C. elegans* ACC were obtained from extracts of fourth-stage larvae expressing (His)_10_-tagged POD-2 (MW ≈ 231 kDa) using Ni-NTA affinity purification, followed by concentration using an ultrafiltration size-exclusion membrane with a 100 kDa cutoff. While enriched in ACC, these preparations also contained other contaminating proteins, as shown below. The partially purified protein extract was employed to monitor the time evolution of the standard reaction mixture containing all reactants required to assay ACC activity (acetyl-CoA, ATP, HCO_3_^*–*^, and Mg^2+^). Typically, after an initial (*t*_0_) HPLC run to separate and quantify the UV-detectable components of the standard reaction mixture, a fixed aliquot of partially purified ACC was added to the reaction batch, and real-time kinetics were monitored by recording HPLC chromatograms of samples taken at regular 24 min intervals over 6 h. Figure 2 shows the chromatograms that were obtained with (Figures 2A and 2B) and without (Figures 2C and 2D) addition of ATP in the reaction mixture.

**Figure 2:**
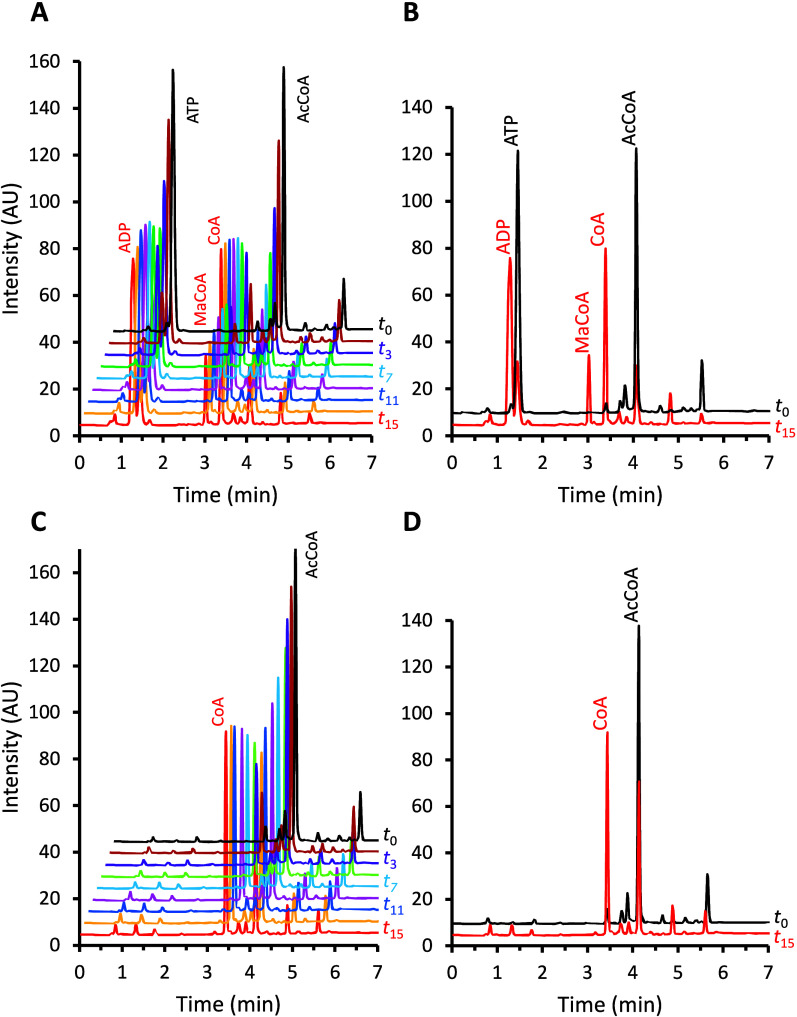
HPLC-based real-time kinetics of ACC activity. Stacked chromatograms of samples without (*t*_0_) and with (*t*_1_–t_15_) the addition of *C. elegans* ACC protein extract to the standard reaction mixture, either with (A, B) or without (C, D) 50 μM ATP, recorded at 24 min intervals over a time course of 6 h. The standard mixture components were 50 μM acetyl-CoA, 50 mM NH_4_HCO_3_, and 10 mM MgCl_2_. A 10 μL aliquot of protein extract, with 2.9 μg/μL protein content (estimated by UV absorption at 280 nm), was added to the reaction batch. (B, D) Superpositions of chromatograms from first (*t*_0_, black) and last (*t*_15_, red) time points. Panel (B) shows a decrease of acetyl-CoA (AcCoA) and ATP and a corresponding increase of malonyl-CoA (MaCoA) and two additional species eluting at 1.28 and 3.46 min, tentatively identified as ADP and free coenzyme A (CoA); panel (D) shows a decrease in AcCoA and an increase of putative free CoA peak at 3.46 min.

The HPLC profiles in the presence of ATP (Figures 2A and 2B) clearly show the decrease of the acetyl-CoA peak and the increase of the malonyl-CoA peak, consistent with the expectation of the net transformation catalyzed by ACC:

$$\mathrm{ATP}+{{\mathrm{HCO}}_{3}}^{-}+\mathrm{acetyl‐CoA}⇆\mathrm{ADP}+P_{i}+\mathrm{malonyl‐CoA}$$

An analogous pattern was also observed for the ATP and flanking ADP peaks, although their proximity to the elution front precluded reliable quantification. The appearance of the malonyl-CoA peak was clearly ATP-dependent, as it was absent without ATP (Figures 2C and 2D). Between the acetyl-CoA and malonyl-CoA peaks, an additional species with an intermediate elution time (3.46 min) also co-evolved with acetyl-CoA consumption in both the presence (Figures 2A and 2B) and absence (Figures 2C and 2D) of ATP. It is inferred that this peak corresponds to free coenzyme A (CoA), and that additional enzymatic activities (other than ACC) that utilize acetyl-CoA and release free CoA must be present in the protein extract.

Proteomic analysis of the *C. elegans* protein extracts and the reacted mixtures reproducibly provided evidence of the composite character of the extracts. For example, a typical proteomic analysis list (Table S2) showed that, out of 136 quantified proteins, the top 16 species (scoring at least 1% molar fraction) cumulatively accounted for 43% of the total assessed molar fraction, with ACC contributing by 1.9% molar (8.1% mass) fraction of the total extract. These figures reveal that affinity purification and subsequent ultrafiltration achieved only a partial purification—typically leading to a final ACC content of 5–10% (w/w)—probably due to the persistence of high molecular weight and aggregated proteins. A further ultrafiltration step increased the relative content of ACC, but led to 1 order of magnitude decrease in recovered protein (not shown). Examination of the pooled protein lists (Table S2) showed that only two enzymes other than ACC could contribute significantly to acetyl-CoA substrate depletion: acetyl-CoA acetyl transferase (ACAT) and acetyl-CoA hydrolase (ACH). ACAT catalyzes the condensation of two acetyl-CoA molecules into acetoacetyl-CoA with free CoA production,^42^ and ACH catalyzes the thioester hydrolysis into free CoA and acetate.^43^ These results further support the assignment of the peak eluting at 3.46 min as free CoA, although it is not possible to rule out contributions of acetoacetyl-CoA (from ACAT catalysis) to the free CoA signal.

Next, a time-course analysis was conducted (Figure 3) comparing the *C. elegans* ACC activity in the absence (Figure 3A) or presence (Figure 3B) of avocadene acetate.^31,32^ Inhibition is evident from the lower levels of malonyl-CoA production and ATP consumption upon addition of the AFA compound (Figures 3A and 3B) and the increased magnitude of inhibition at higher AFA concentrations (Figure 3C). Notably, while the depletion of acetyl-CoA appeared largely unaffected by the inhibitor, the production of free CoA increased, suggesting that ACC inhibition was balanced by a relative increase in the activity of ACAT and ACH present in the *C. elegans* extracts. This conclusion was further supported by comparing the time evolution of the AcCoA substrate and free CoA in the presence vs absence of ATP (Figure 3D): while consumption of acetyl-CoA decreased in the absence of ATP, the accumulation of CoA was nearly invariant, again implicating residual activity of ACAT and ACH in the partially purified protein extracts (also see Figure 2D and Figure S1). Qualitatively speaking, these observations suggest that avocadene acetate has no inhibitory effect on ACAT and ACH activity.

**Figure 3:**
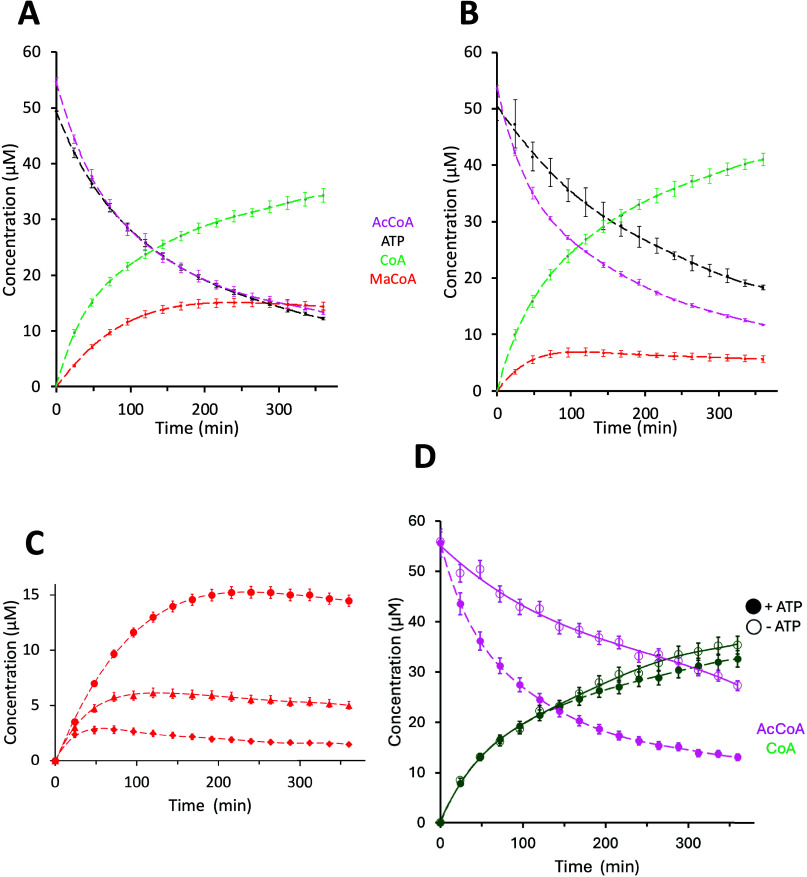
Time course of ACC substrate and product concentrations under different conditions, as measured by HPLC. (A, B) Acetyl-CoA, ATP, CoA, and malonyl-CoA evolution in the absence (panel (A)) and presence (panel (B)) of 50 μM avocadene acetate, an ACC inhibitor. Plotted data are means of three replicates with error bars of ±1 SD, measured under the same conditions as reported in Figure 2 caption. (C) Time course of malonyl-CoA concentration produced with starting acetyl-CoA concentration of 50 μM, in the absence (circles) and presence of avocadene acetate at inhibitor-to-substrate ratios of 1:1 (triangles) and 2:1 (diamonds). (D) Depletion of acetyl-CoA and formation of CoA in the absence (empty circles) and presence (filled circles) of ATP. Plotted data in panels (C) and (D) represent single determinations with errors equal to average concentration uncertainties. Dashed lines in all panels are polynomials drawn as eye guides; fitting parameters of malonyl-CoA data shown in panels (A) and (B) are reported in Table S3.

### NMR-Based Real-Time Kinetics of ACC

As previously reported,^31^ direct observation of malonyl-CoA in D_2_O by NMR is problematic, because the keto–enol tautomerism involving the malonyl moiety leads to the loss of the methylene signal due to isotopic exchange. Unfortunately, operating in H_2_O, to prevent signal cancellation, is quite impractical for real-time quantitative experiments. Therefore, for the sake of uniform signal-to-noise ratios and reproducible results, it is much better to operate in D_2_O. Under this condition, it is possible, instead, to exploit the resolution of the CoA thio-methylene signal to inspect the onset of malonyl-CoA. An analogous corresponding signal is also observable for acetyl-CoA, though this species is much more readily (and better) estimated from the terminal acetyl methyl peak. Figure 4A shows that NMR spectra of the corresponding standards (green traces) match well the superimposed traces of the initial (black) and final (red) spectra for the ACC-catalyzed conversion of acetyl-CoA to malonyl-CoA. The lower intensity of the growing malonyl-CoA signal relative to the starting acetyl-CoA counterpart is consistent with the presence of additional enzyme activities in the *C. elegans* extracts that also consume the acetyl-CoA substrate, as inferred from HPLC-based kinetics and proteomics results (Table S2). Specific experiments were conducted to further characterize the alternative sources of acetyl-CoA depletion, as reported in Figure S2 and Note S1. An additional aspect of the NMR assessment was the non-negligible contributions from the *C. elegans* protein extract to the NMR regions used for integration of the growing malonyl-CoA thio-methylene and free acetate methyl, as documented in Figure 4B. The corresponding integrals were therefore corrected by the amount determined from the isolated extract.

**Figure 4:**
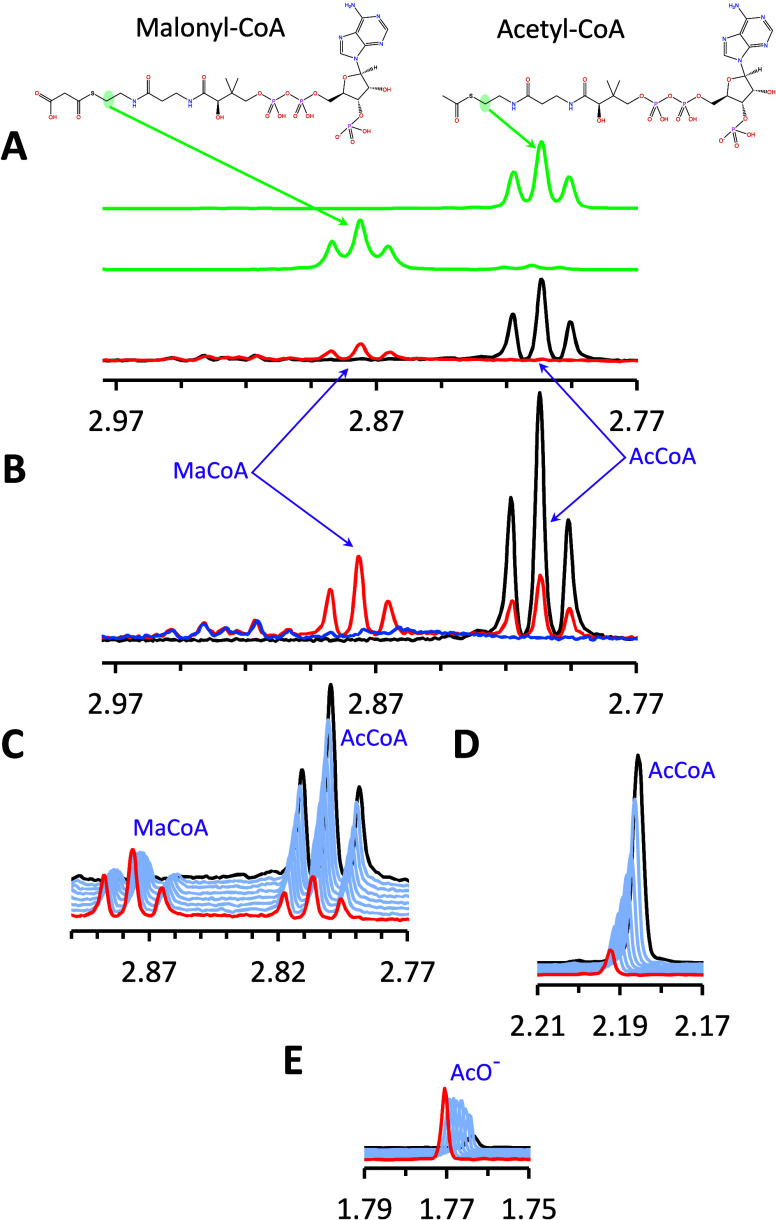
Traces of relevant regions of ^1^H NMR spectra with evidence of an ACC-catalyzed reaction (chemical shifts are in ppm). (A) Upper two traces (green): spectra of acetyl-CoA and malonyl-CoA standards, showing resolution of the thio-methylene signals (highlighted in corresponding chemical structures). Bottom traces: superimposed spectra of the same region in the initial (black) and final (red) sampling of the reaction mixture. The conversion of acetyl-CoA into malonyl-CoA shows the same pattern as the standards. For this sample, initial and final acquisitions were at 7.6 min and ∼32 h after addition of ACC extract. (B) Superposition of ^1^H NMR spectra of the reaction mixture before (black) and ∼13 h (red) after addition of extract vs extract only (blue), illustrating background intensity deriving from the extract components. (C–E) Stacked plots of ^1^H NMR spectral regions showing the decrease and the increase of thio-methylene pseudotriplets, respectively from acetyl-CoA (∼2.80 ppm) and malonyl-CoA (∼2.87 ppm) (panel (C)); the decrease of the acetyl-CoA acetyl methyl singlet (panel (D)); and the increase of the free acetate (AcO^–^) methyl singlet (panel (E)). Except for acetyl-CoAin panel (A) (81.6 μM), NMR sample compositions are listed in the Experimental Section.

The typical time course of NMR kinetics in Figures 4C–E illustrates substrate consumption, product formation, and accumulation of free acetate. The corresponding time courses of the acetyl-CoA and malonyl-CoA concentrations are presented in Figure 5. The comparison of data collected in the absence (Figure 5A) and presence (Figure 5B) of 25 μM avocadene acetate clearly shows the reduction of malonyl-CoA accumulation. When substrate and (side) product concentrations are measured in the absence of ATP (Figure 5C), no malonyl-CoA is formed, as expected, while the consumption of acetyl-CoA and the production of free acetate from non-ACC activities are still observed.

**Figure 5:**
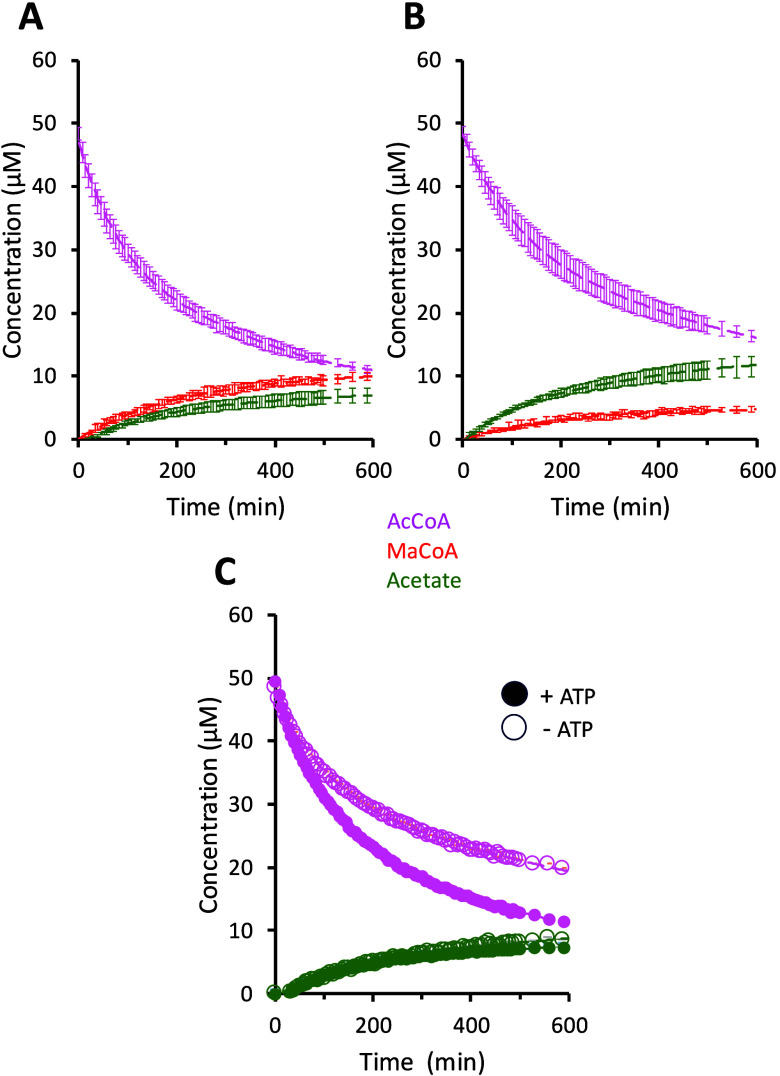
Time evolution of acetyl-CoA, acetate, and malonyl-CoA concentrations under different conditions, measured by NMR. (A, B) Concentration trends in the absence (panel (A)) and presence (panel (B)) of 25 μM avocadene acetate. Contrary to malonyl-CoA, the acetate concentration is independent of ACC, whereas acetyl-CoA concentration depends on all three enzyme activities (ACC, ACAT, ACH) that are present in the *C. elegans* protein extract. All plotted data are averages of triplicate measurements, with standard deviations reported as error bars. (C) Acetyl-CoA depletion and acetate formation in the presence (filled symbols) and absence (empty symbols) of ATP. In any plot the dashed lines are polynomials drawn as eye guides. The fitting parameters of malonyl-CoA data in panels (A) and (B) are reported in Table S3.

Despite the qualitative analogy between NMR (Figures 5A and 5B) and HPLC (Figures 3A and 3B) trends, even at 1:0.5 substrate-to-inhibitor ratio, the NMR time-course curves systematically displayed reduced changes in evolved levels of reactants and products, suggesting lower concentrations of ACC and other enzymes in the batch of *C. elegans* protein extract. In fact, NMR samples with 1:1 substrate-to-inhibitor ratio showed no detectable formation of malonyl-CoA.

In the absence of a proteomic assessment of the extracts used for the NMR measurements, the initial slopes of acetyl-CoA and malonyl-CoA concentration progress were estimated using the data recorded within 48 min from extract addition in the absence of inhibition (Figure 5A). Assuming linearity in that interval, the comparison with the corresponding region of the HPLC-monitored kinetics (Figure 3A) showed that the activity loss of the protein pool consuming acetyl-CoA and the activity loss of ACC producing malonyl-CoA were 22% and 69%, respectively. The estimated ACC activity loss translates into an ACC mass percentage of 2.5% in the extract used for NMR (from 8.1% of the extract used for HPLC). This estimated ACC content was employed to evaluate the enzyme *V*_max_ values from the NMR kinetics data.

### Kinetic Parameter Determination

The qualitative analysis illustration and the proteomic evidence show that acetyl-CoA is not only the substrate of the carboxyl transfer catalyzed by ACC, but also the substrate of two other enzymes, ACAT and ACH. The contributions of these two activities to the acetyl-CoA decay cannot be modeled from CoA and acetate accumulation without additional independent information because the condensation (ACAT) and hydrolysis (ACH) processes occur with different kinetic orders and rates with respect to acetyl-CoA. Nor are the evolutions of ATP and ADP concentrations available to characterize the biotin carboxylation step of the ACC reaction, under pseudo-first-order conditions implied by high bicarbonate concentration, due to the limited quantitative reliability of signals close to the elution front. Therefore, the only profile that may be exploited to obtain ACC kinetic parameters is the malonyl-CoA concentration progress, which can provide the relevant kinetic parameters even in the presence of other enzyme activities that employ the same substrate as the ACC-catalyzed process.

Typically, the enzyme kinetics determinations are conducted by measuring the initial reaction rates of substrate consumption and/or product formation at several substrate concentrations, which are then used to construct the double reciprocal plot^44^ and estimate the Michaelis–Menten constant (*K*_M_) and the maximum velocity (*V*_max_) values,^45^ under conditions of steady-state approximation validity.^46^ This method is often quite demanding in terms of experimental effort and the required materials. For ACC in particular, all the kinetics studies to date have been carried out using the initial rate method, with the vast majority of the determinations based on ^14^C-labeled substrates and products.^30,33–37^

Provided spectroscopic or chromatographic techniques and suitable experimental settings are available for real-time measurements of the concentration progress curves for substrate(s) and/or product(s), an alternative method can be applied using a single initial concentration of the substrate(s).^47^ The Michaelis–Menten equation expresses the reaction rate (*v*) in terms of time (*t*) dependence of the substrate concentration ([*S*]) and fixed parameters (*K*_M_ and *V*_max_):

$$v=-\frac{d\left[S\right]}{dt}=V_{\max}\frac{\left[S\right]}{\left[S\right]+K_{M}}$$

This expression can be integrated, and the corresponding implicit solution can be made explicit^48^ by introducing the Lambert *W* function^49^ (see the SI (Note S2)). The resulting new solution

$$\left[S\right]=K_{M}W\left(x\right)$$

where the Lambert *W* function independent variable (*x*) cumulates time (*t*) and kinetic parameters ([*S*_0_], *K*_M_, and *V*_max_), and can be approximated by analytical expressions^50,51^ (see the SI (Note S2)), thereby enabling direct fitting of experimental data.

The progress curves of the malonyl-CoA concentration obtained from the experimental chromatographic and NMR profiles were thus fitted, and the values of *K*_M_ and *V*_max_ could be determined in the absence and presence of avocadene acetate (Table 1). The details of the fitting are reported in the SI (Table S3 and Note S2).

**Table 1:** *C. elegans* ACC Kinetic Parameters from Malonyl-CoA Concentration Time Progress^a^

*T* (°C)	*K*_M_ (μM)	*V*_max_ (μM min^–1^ mg^–1^)	inhibitor/monitoring
25	7.5 ± 1.4	(1.0 ± 0.1) × 10^2^	no inhibitor/HPLC
25	1.5 ± 0.5	80 ± 13	+ avocadene acetate/HPLC
20	4.8 ± 0.7	23 ± 1	no inhibitor/NMR
20	3.0 ± 0.3	19 ± 5	+ avocadene acetate/NMR

a All reported *K*_M_ values refer to acetyl-CoA substrate. As explicitly indicated by the units, the *V*_max_ values are given per milligram of enzyme. For the HPLC-based estimates, the *V*_max_ values obtained from the fitting were scaled to 1 mg of enzyme based on the overall protein content (2.9 μg/μL) and the ACC mass percentage (8.1%) of the experimental protein extract samples. For the NMR-based estimates, the same procedure was adopted using the proper protein content of the employed extract (1.72 μg/μL) and assuming a value of 2.5% for the ACC mass percentage, as obtained from the initial slopes of the concentration evolution (see text). The listed kinetic parameters of *C. elegans* ACC obtained in this work are averages ± standard deviations of triplicate determinations. The listed kinetic parameters are averages ± standard deviations, obtained from the fitting of data collected in triplicate at substrate/inhibitor ratio 1:0 and 1:1 (HPLC) or 1:0 and 1:0.5 (NMR). No fitting was attempted, instead, for the data recorded at ratio 1:2 (HPLC), because only a single time progress curve was available. The corresponding trend was, however, qualitatively consistent with the previous body of evidence, as shown in Figure 3C.

The results in Table 1 concerning only the malonyl-CoA profiles show that there is excellent agreement among values obtained from HPLC and NMR data. The two sets of *K*_M_ values are only slightly different, and their 95% confidence intervals are largely coincident. Although *K*_M_ is theoretically independent of the enzyme concentration, some difference is to be expected, due to the lower temperature of the NMR determinations (20 °C), compared with the HPLC conditions (25 °C). On the other hand, the *V*_max_ values consistently diverge, because of the different ACC concentration in HPLC and NMR samples, besides the contribution from the temperature difference. Overall, the values here determined for *C. elegans* ACC are broadly consistent with previously reported values (Table S4). The variation of the enzyme *K*_M_ and *V*_max_ values induced by avocadene acetate reveals a decrease in both kinetic parameters for either HPLC- or NMR-based estimates. A decrease in the *V*_max_ value is always expected in the presence of an inhibitor. In contrast, a reduction of *K*_M_ represents the typical counterintuitive increase of affinity that characterizes the uncompetitive inhibition, whereby the inhibitor binds exquisitely to the catalytic complex between the enzyme and the substrate.

## Discussion

The results that were obtained from the kinetic assessment of *C. elegans* ACC activity are noteworthy for two aspects. First, the experimental design was different, with respect to the vast majority of the previous ACC kinetic studies. The traditionally adopted strategy for those studies relied on ^14^C-labeled substrates and products.^30,33–37^ Accurate double reciprocal plots required critical amounts of radioactivity counting acquisitions to determine initial rates at different substrate concentrations.^44^ Indirect quantifications of ATP consumption through NADH^52^ or malonyl-CoA onset through malonyl-CoA reductase^53^ have also been used, in alternative or as a complement to liquid scintillation, most often with pure enzymes (recombinant or purified), and less frequently with mixtures.^54^

The methodology has been simplified in the present approach, thereby reducing the experimental efforts. The measurements were carried out by real-time UV monitoring of chromatographic profiles or directly in the intact reaction mixtures by NMR, at a single substate concentration. The resulting progress curves could be directly employed to extract *K*_M_ and *V*_max_, thanks to an established method^47–51^ for accurate data fitting by analytical solutions of the integrated Michaelis–Menten equation. The data sets came from partially purified preparations that contained other enzymes competing for the ACC substrate and presented a challenge to the NMR analysis because the spectral integration required correction for the background contribution.

The other interesting aspect of the present work is related to the characterization of the inhibition brought about by avocadene acetate to ACC activity. The picture emerging from the interpretation of the changes in the *K*_M_ and *V*_max_ values that are observed in the presence of the inhibitor mirrors the pattern of uncompetitive inhibition that strictly speaking corresponds to the binding of the inhibitor to the enzyme–substrate complex.

A previous work by Hashimura and colleagues^32^ first reported the inhibitory effect of avocadene acetate on rat liver ACC with an estimated IC_50_ value of 9.4 μM. The Cheng and Prusoff equation links the IC_50_ value (the total concentration of inhibitor necessary to decrease the enzyme activity by 50%) to the substrate concentration ([*S*]), and to the kinetic and inhibition parameters of the enzyme according to eq 1:^55^

1 $$K_{i}=\frac{{\mathrm{IC}}_{50}}{\left(1+\frac{\left[S\right]}{K_{M}}\right)}$$

where *K*_*i*_ is the inhibition constant corresponding to the thermodynamic dissociation constant of the inhibitor-enzyme complex. The same inhibition constant also accounts for the apparent variation of the *K*_M_ value that changes into *K*_M_^app^ when measured in the presence of an inhibitor at concentration [*I*]:

2 $$K_{M}^{\mathrm{app}}=\frac{K_{M}}{\left(1+\frac{\left[I\right]}{K_{i}}\right)}$$

From the HPLC-based data in Table 1, using [*I*] = 50 μM and eq 2, a *K*_*i*_ value of 12.5 μM is estimated that should be entered in eq 1 to evaluate IC_50_. The value to be used for [*S*] in eq 1, however, cannot be that of the acetyl-CoA total concentration, due to the other enzymes in the employed extract, which compete for the same substrate. Based on the fitting of the progress curve data, the effective substrate concentration consistent with the experimental ACC activity is ∼15 μM (SI (Table S3)). With this value, eq 1 returns a result of 37 μM for IC_50_, i.e., about four times the measured 9.4 μM value.^32^ If the NMR-based parameters are used, an IC_50_ of 45 μM is obtained, i.e., about five times the reported value. These estimates are affected by quite substantial errors (70%–80%) and their significance is, therefore, limited to the magnitude order. Moreover, comparing the calculated IC_50_ values for *C. elegans* ACC to experimentally determined values for a homologous enzyme does not provide insight into the actual type and mechanism of inhibition.

A more insightful tool could be the refinement of the avocadene acetate docking to the *C. elegans* ACC complex based on our former modeling of the same assembly.^31^ In that modeled ACC/POD-2 dimer, the CT domain active site (fragment 1438–2165) shares a high degree of structural similarity (rmsd <3 Å) to the solved yeast structures^14,40^ (PDB ID: 5csl and 1w2x). Induced-fit docking, which allows sampling of protein target site and ligand conformations,^41^ predicted that avocadene acetate binds to the CT active site without any substrate with an affinity of 2.5 μM (Figure 6).^31^ In the absence of solved crystal structures for ACC–acetyl-CoA complexes, the available coordinates of yeast ACC-CoA complex^14^ (PDB ID: 5csl) were used to model the interactions of avocadene acetate with the enzyme and the quasi-substrate complex. The docking to the ACC/POD-2 CT active site hosting CoA produced a complex with ligand affinity of 4.0 μM. Within the accuracy of the methodology, the previous and the last docking results indicate that avocadene acetate binding to the ACC/POD-2 CT active site is essentially unaffected by the presence of CoA. In both scenarios, in fact, the avocadene acetate binding mode remains similar, with its acetyl extremity in close proximity to the position occupied by the terminal cysteamine moiety of CoA, when present (Figure 6).

**Figure 6:**
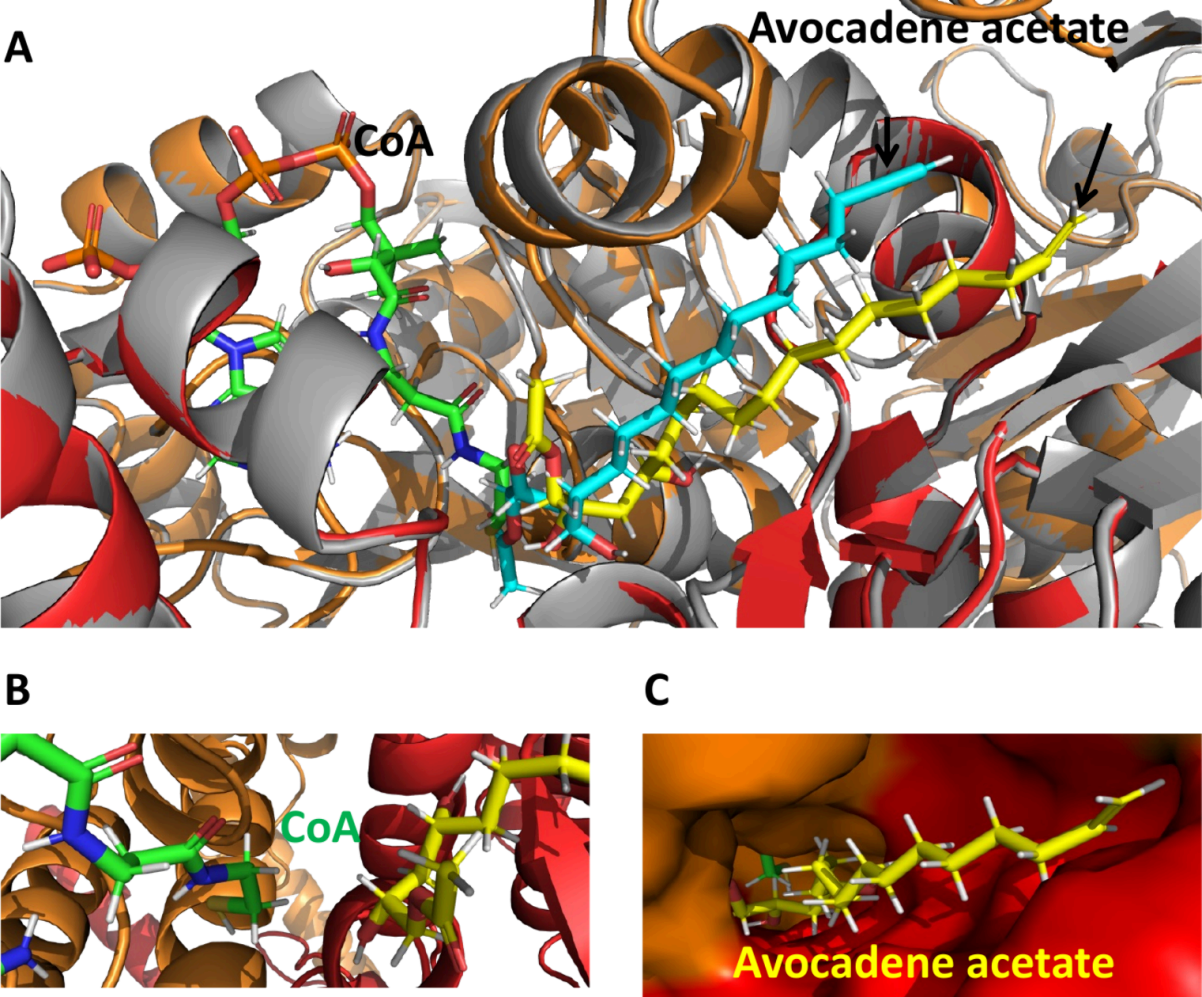
Docking of avocadene acetate to the CT domain active site of *C. elegans* ACC. (A) The docked inhibitor arrangement is shown in the presence (yellow chain) and absence (cyan chain) of CoA. In both cases, the acetyl group of avocadene acetate binds close to the position occupied by the terminal cysteamine moiety of CoA (when present). The computed avocadene acetate affinities, in the presence or absence of CoA, are quite similar, i.e., 4.0 or 2.5 μM, respectively, with a difference within the uncertainty of the calculation method. (B) CoA is spatially close to avocadene acetate (minimal distance 0.24 nm), but there are no hydrogen bonds between them. (C) Surface rendering highlights the proximity of avocadene acetate and CoA in the CT active site tunnel.

Using eq 1 with the same [*S*] values as employed for the previous IC_50_ calculation (Table S3) and the modeled CT-site complex lower (higher) predicted *K*_d_ value—corresponding to *K*_*i*_ – would lead to IC_50_ = 7.5 (12) μM or 7.1 (11) μM, after imposing the *K*_M_ values obtained from HPLC or NMR data, respectively (Table 1). This proves to be in quite satisfactory agreement with the value reported for rat liver ACC.^32^

More significantly, however, visual inspection of the modeled interaction provides crucial clues for the inhibition mechanism (Figure 6). Addressing the CT site is not in contrast with the uncompetitive nature of the avocadene acetate inhibition, which was inferred from the kinetic parameters of Table 1. The model of Figure 6 shows that the inhibitor binds at a different location in the catalytic domain with respect to the position of the substrate. Avocadene acetate in fact hinders the proper positioning of carboxybiotin to accomplish the carboxyl transfer, which features an arrangement that is consistent with uncompetitive inhibition.

Since the computed dissociation constants of avocadene acetate from the ternary complex with the ACC/POD-2–CoA (4.0 μM) is not smaller than the corresponding parameter for the binary complex with ACC/POD-2 (2.5 μM), as expected from a pure uncompetitive inhibition scheme, a mixed uncompetitive/noncompetitive pattern may be hypothesized which could be compatible or, at least, not inconsistent with the uncompetitive pattern signature implied by the *K*_M_ values of Table 1. In essence, although some care is necessary to conclusively validate the results of homology modeling conducted with templates, which may partially deviate with respect to *C. elegans* ACC, the convergence of the experimental results and the modeling expectations is quite satisfactory and encourages further investigation along the same lines.

In conclusion, the results presented here demonstrate that a radically different approach, which does not require the demanding purification protocols and expensive substrate radiolabeling of previous methods used to study ACC kinetics, can be successfully adopted to obtain reliable kinetic parameters and provide valuable insights into the mechanisms of enzyme catalysis and inhibition. Since ACC is increasingly recognized as a crucial metabolic crosspoint for energy metabolism and could represent a potential therapeutic target for obesity, cancer, and hepatic steatosis, the availability of reliable and simpler methods than earlier ones for kinetic studies is extremely important.

## Supplementary Material



## Notes

Tables reporting (i) chromatographic assessment calibrations; (ii) MASCOT proteomic analysis; (iii) kinetic fitting parameters of concentration time-course data; (iv) comparison of kinetic parameters; Figures illustrating (i) acetyl-CoA depletion and CoA formation; (ii) ACC-independent consumption of acetyl-CoA. Explicative notes for (i) ACC-independent consumption of acetyl-CoA; (ii) derivation of the fitting equation for kinetic data (PDF)
