# Supplementary material for: Assessing Acetyl-Coenzyme A Carboxylase Activity and Inhibition in Caenorhabtidis elegans Using a Partially Purified Protein Extract
Source: Anal Chem. 2025 Aug 26;97(35):18907–17. doi: 10.1021/acs.analchem.5c00828 (PMC12435777; doi:10.1021/acs.analchem.5c00828)
Supplement: Supplementary file 1 [file ac5c00828_si_001.pdf]

Assessing acetyl-coenzyme A carboxylase activity and inhibition in *Caenorhabtidis elegans* using a partially purified protein extract

Gennaro Battaglia,[1,2‡] Yamanappa Hunashal,[1‡] Suma Gopinadhan,[3‡] Hin Hark Gan,[4] Yasmine Moussa,[3] Fathima S. Mohammed Refai,[3,5] Angela Amoresano,[2,6] Hala Zahreddine Fahs,[4] Kristin C. Gunsalus,[3,4]* Gennaro Esposito,[1,6]* Fabio Piano,[1,3,4]*

1 Science Division, New York University Abu Dhabi, Saadiyat Island, Abu Dhabi, United Arab Emirates

2 Dipartimento di Scienze Chimiche, Università di Napoli “Federico II”, 80138 Naples, Italy

3 Center for Genomics and Systems Biology, New York University Abu Dhabi, Saadiyat Island, Abu Dhabi, United Arab Emirates

4 Center for Genomics and Systems Biology, Department of Biology, New York University, New York, NY, USA

5 Dipartimento di Medicina, Università di Udine, 33100 Udine, Italy

6 Istituto Nazionale Biostrutture e Biosistemi, 00136 Rome, Italy

‡Equal contribution

* Corresponding authors. Email: rino.esposito@nyu.edu; kcg1@nyu.edu; fp1@nyu.edu

SUPPORTING INFORMATION

SUPPLEMENTARY EXPERIMENTAL SECTION

Chemicals and reagents. Urea, 4-(2-Hydroxyethyl)-1-piperazine ethanesulfonic acid (HEPES), dithiothreitol (DTT), tris(2-carboxyethyl)-phosphine (TCEP), trypsin, iodoacetamide (IAM), ammonium bicarbonate (NH_4HCO_3) magnesium dichloride ($MgCl_2$), , deuterium oxide (D_2O), glycerol, imidazole, acetyl-CoA and malonyl-CoA standards were purchased from Sigma-Merck. Protease inhibitor cocktail Halt was from Thermo Scientific. Ultra-pure adenosine triphosphate (ATP) came from Promega (Madison, WI, USA). Formic acid (HCOOH), methanol (CH3OH) and acetonitrile (CH3CN) were from J.T. Baker (Phillipsburg, New Jersey, USA). ZipTips with 0.6 µL C18 resin (Sigma-Merck) were used for desalting and concentrating peptides. Synthetic avocadene acetate ((2R,4R)-2,4-dihydroxyheptadec-16-en-1-yl acetate) was purchased from ChiroBlock GmbH (Wolfen, Germany). The other deuterated products used for the NMR sample preparations, i.e. CD_3CN, tris(hydroxymethyl)aminomethane-d11 (Tris-d11), and ethylene glycol-d6, were all obtained from Cambridge Isotopes Laboratories (Tewksbury, MA, USA).

HPLC system. An Agilent 1200-series high-performance liquid chromatography (HPLC) unit (Agilent Technologies, Santa Clara, USA) consisting of a binary pump, autosampler, temperature-controlled column compartment, and diode array detector (DAD) was used. HPLC run were carried out using an Infinity Lab Poroshell 120 EC-C18 column (3.0x150mm; 2.7 µm; Agilent Technologies). After selecting the detection wavelength, the column temperature was set at 25 °C with a flow rate of 0.8 mL/min. The injection volume was 10 µL.

LC-Orbitrap instrumentation and conditions. The liquid chromatography (LC) instrument linked to the mass spectrometer detector was an Ultimate U3000 Nano LC System (Dionex) fitted with a C18 trap (Pep Map nano Viper, Thermo Scientific) and a resolving column (PepMap RSLC) with inner diameters of 75 µm, particle size 2 µm and lengths of 50 cm (75 µm, 500 mm, 2 µm) and working at 400 nL/min. flow rate. The mobile phases were aqueous solutions with 0.1% HCOOH and 2% CH_3CN (solvent A), and with 0.08% HCOOH and 80% CH_3CN (solvent B). The samples were loaded in solvent A and eluted as follows: 1-45 min., linear gradient from 0 to 35% B; 45-50 min., linear gradient from 35 to 60% B; 50-60 min., constant 60% B; 60-61 min., linear gradient from 60 to 0% B; 61-75 min., constant 0% B (initial condition); 75-80 min., re-equilibration at 0% B, with solvent A always complementing to 100%. The LC system was coupled to an LTQ-Orbitrap Velos mass spectrometer (Thermo Scientific) equipped with an Easy Spray ion source and operated in positive-ion mode. The spray voltage was 2kV and the full scans were acquired in a Fourier transform MS mass analyser over m/z 350–1800 Th at a resolution of 60,000. The MS/MS analyses were performed under data-dependent mode to fragment the top 5 precursors using collision-induced dissociation.

Preparative protein assay. The solutions that had used for chromatographic kinetic assessments were sometime submitted to gel-free proteomics. The proteins were quantified by Bradford assay. A sample volume corresponding to a specific amount of protein content (50 µg) was used to carry out the proteolytic digestion. Proteins in 6 M urea were first reduced with 20 mM DTT and then incubated for 45 min. at 95 °C. After cooling down the protein solution to room temperature, the cysteines were alkylated by the addition of IAM 40 mM, followed by incubation in the dark for 30 min. The excess IAM was quenched with 20mM DTT for 60 min. in the dark. Before adding the protease, urea was diluted to a concentration of 1 M. Trypsin was added to reach 1:50 enzyme/protein ratio and incubated for 18 hours at 37 °C. Digestion was stopped by adding 2% HCOOH. ZipTip with 0.6 µL composed of C18 resin was used for sample desalting prior to mass spectrometry analysis. The dried sample was dissolved in 50 µL of aqueous solvent containing 2% CH_3CN and 0.2% HCOOH and analysed by LC-MS/MS Orbitrap.

Protein identification by MASCOT. The raw MS/MS data were analyzed using MASCOT algorithm (Mascot 2.4.0, Matrix Science, London,UK) against the UniProt *C. elegans* database. The precursor (10 ppm) and fragment mass tolerances (0.5 Da) were set. The protease specificity used to generate the digestion peptides was set for trypsin (cleavage at the C-terminus of Lys and Arg residues) along with maximum missed cleavages value of 3. Carbamidomethyl on cysteine as fixed modification, oxidation of methionine and formation of pyro-Glu from N-terminal Glu or Gln were considered as variable modifications for database search.

Table S1 – Chromatographic assessment calibration data*

Acetyl-CoA								
µM	Response 1	Response 2	Response3	Average	St Dev	rsd%	LOD	LOQ
4.68	37.5	26.8	35.0	33.1	5.6	16.9	0.5	1.6
9.37	70.6	70.1	69.5	70.1	0.6	0.8		
18.75	135.1	135.8	133.0	134.6	1.4	1.1		
37.5	262.2	260.4	259.1	260.6	1.5	0.6		
75	512.7	509.5	505.7	509.3	3.5	0.7		
150	963.2	950.4	955.2	956.3	6.4	0.7		

Malonyl-CoA								
µM	Response 1	Response 2	Response3	Average	St Dev	rsd%	LOD	LOQ
4.68	29.4	29.2	28.8	29.1	0.3	1.0	0.3	1.0
9.37	57.2	57.1	56.7	57.0	0.3	0.4		
18.75	112.0	111.9	111.0	111.6	0.5	0.5		
37.5	220.1	219.0	217.7	218.9	1.2	0.6		
75	433.4	431.2	430.1	431.6	1.7	0.4		
150	836.1	828.2	830.2	831.5	4.2	0.5		

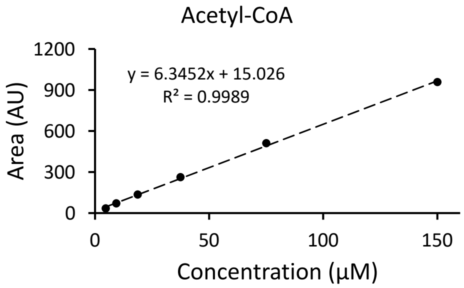

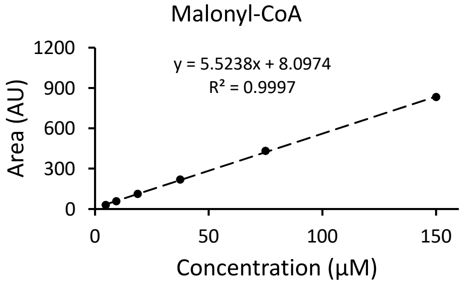

***** The linearity range and the limits of detection (LOD) and quantification (LOQ) were checked before performing the kinetic measurements. The tests were conducted under the same conditions as the kinetic assays while also taking into account a possible matrix effect. All standard solutions were in fact prepared in 50 mM NH_4HCO_3 and 10 mM $MgCl_2$. The solutions of malonyl-CoA and acetyl-CoA spanning a concentration range 5 -150 µM were prepared in triplicate.

Table S2 – MASCOT Proteomic analysis*

prot_acc	prot_desc	emPAI	%mol-Prot	prot_cover	prot_sequences_sig
MYSP_CAEEL	Paramyosin OS=Caenorhabditis elegans OX=6239 GN=unc-15 PE=1 SV=2	18.61	7.8	75.5	74
METK1_CAEEL	Probable S-adenosylmethionine synthase 1 OS=Caenorhabditis elegans OX=6239 GN=sams-1 PE=1 SV=1	17.27	7.2	73.9	29
EF1A_CAEEL	Elongation factor 1-alpha OS=Caenorhabditis elegans OX=6239 GN=eft-3 PE=3 SV=1	15.69	6.6	73	35
HSP7A_CAEEL	Heat shock 70 kDa protein A OS=Caenorhabditis elegans OX=6239 GN=hsp-1 PE=1 SV=2	14.69	6.1	69.4	46
THILH_CAEEL	Acetyl-CoA acetyltransferase homolog, mitochondrial OS=Caenorhabditis elegans OX=6239 GN=kat-1 PE=1 S	4.97	2.1	58.7	19
H2L0M0_CAEEL	Acetyl-CoA carboxylase OS=Caenorhabditis elegans OX=6239 GN=pod-2 PE=1 SV=1	4.56	1.9	62.3	21
GCP_CAEEL	Bifunctional glyoxylate cycle protein OS=Caenorhabditis elegans OX=6239 GN=icl-1 PE=1 SV=2	4.22	1.8	57	47
MYO4_CAEEL	Myosin-4 OS=Caenorhabditis elegans OX=6239 GN=unc-54 PE=1 SV=2	2.97	1.2	49.9	82
HSP7F_CAEEL	Heat shock 70 kDa protein F, mitochondrial OS=Caenorhabditis elegans OX=6239 GN=hsp-6 PE=1 SV=2	2.71	1.1	49.9	27
METK3_CAEEL	Probable S-adenosylmethionine synthase 3 OS=Caenorhabditis elegans OX=6239 GN=sams-3 PE=1 SV=1	2.63	1.1	45	16
CH60_CAEEL	Chaperonin homolog Hsp-60, mitochondrial OS=Caenorhabditis elegans OX=6239 GN=hsp-60 PE=2 SV=2	2.58	1.1	49.5	24
EF1B1_CAEEL	Probable elongation factor 1-beta/1-delta 1 OS=Caenorhabditis elegans OX=6239 GN=eef-1B.1 PE=1 SV=1	2.46	1.0	45.1	8
TDX1_CAEEL	Probable peroxiredoxin prdx-3 OS=Caenorhabditis elegans OX=6239 GN=prdx-3 PE=3 SV=1	2.45	1.0	50.9	10
EF1B2_CAEEL	Probable elongation factor 1-beta/1-delta 2 OS=Caenorhabditis elegans OX=6239 GN=eef-1B.2 PE=1 SV=4	2.42	1.0	65.4	10
METK4_CAEEL	Probable S-adenosylmethionine synthase 4 OS=Caenorhabditis elegans OX=6239 GN=sams-4 PE=1 SV=1	2.39	1.0	44.6	15
SAHH_CAEEL	Adenosylhomocysteinase OS=Caenorhabditis elegans OX=6239 GN=ahcy-1 PE=3 SV=1	2.31	1.0	53.5	18
GMPR_CAEEL	GMP reductase OS=Caenorhabditis elegans OX=6239 GN=F32D1.5 PE=3 SV=1	2.12	0.9	31.6	12
RPIA_CAEEL	Probable-ribose 5-phosphate isomerase OS=Caenorhabditis elegans OX=6239 GN=rpia-1 PE=3 SV=1	2.11	0.9	41.8	10
PDI2_CAEEL	Protein disulfide-isomerase 2 OS=Caenorhabditis elegans OX=6239 GN=pdi-2 PE=1 SV=1	2	0.8	38.7	19
TBA2_CAEEL	Tubulin alpha-2 chain OS=Caenorhabditis elegans OX=6239 GN=tba-2 PE=1 SV=1	1.93	0.8	46.2	15
SYDC_CAEEL	Aspartate--tRNA ligase, cytoplasmic OS=Caenorhabditis elegans OX=6239 GN=dars-1 PE=3 SV=1	1.9	0.8	45.8	17
ALF2_CAEEL	Fructose-bisphosphate aldolase 2 OS=Caenorhabditis elegans OX=6239 GN=aldo-2 PE=2 SV=1	1.88	0.8	39.6	12
IF5A2_CAEEL	Eukaryotic translation initiation factor 5A-2 OS=Caenorhabditis elegans OX=6239 GN=iff-2 PE=2 SV=1	1.77	0.7	36.6	5
KARG1_CAEEL	Probable arginine kinase F46H5.3 OS=Caenorhabditis elegans OX=6239 GN=F46H5.3 PE=3 SV=2	1.72	0.7	49.5	13
TCPE_CAEEL	T-complex protein 1 subunit epsilon OS=Caenorhabditis elegans OX=6239 GN=cct-5 PE=2 SV=1	1.63	0.7	32.3	17
ENO_CAEEL	Enolase OS=Caenorhabditis elegans OX=6239 GN=enol-1 PE=1 SV=3	1.6	0.7	39.2	12
DEOC_CAEEL	Putative deoxyribose-phosphate aldolase OS=Caenorhabditis elegans OX=6239 GN=F09E5.3 PE=3 SV=1	1.58	0.7	50.8	10
EF1G_CAEEL	Probable elongation factor 1-gamma OS=Caenorhabditis elegans OX=6239 GN=eef-1G PE=1 SV=1	1.54	0.6	31.9	11
SUCA_CAEEL	Succinate--CoA ligase [ADP/GDP-forming] subunit alpha, mitochondrial OS=Caenorhabditis elegans OX=623	1.53	0.6	35.7	8
ADH1_CAEEL	Alcohol dehydrogenase 1 OS=Caenorhabditis elegans OX=6239 GN=sodh-1 PE=2 SV=2	1.5	0.6	40.7	10
ACT1_CAEEL	Actin-1 OS=Caenorhabditis elegans OX=6239 GN=act-1 PE=1 SV=1	1.48	0.6	41.8	11
TCPQ_CAEEL	T-complex protein 1 subunit theta OS=Caenorhabditis elegans OX=6239 GN=cct-8 PE=1 SV=3	1.47	0.6	43.2	17
MMSA_CAEEL	Probable methylmalonate-semialdehyde dehydrogenase [acylating], mitochondrial OS=Caenorhabditis elegan	1.46	0.6	42.3	15
TBB2_CAEEL	Tubulin beta-2 chain OS=Caenorhabditis elegans OX=6239 GN=tbb-2 PE=1 SV=1	1.41	0.6	61.1	14
EF2_CAEEL	Elongation factor 2 OS=Caenorhabditis elegans OX=6239 GN=eef-2 PE=1 SV=4	1.32	0.6	37.7	24
MLR1_CAEEL	Myosin regulatory light chain 1 OS=Caenorhabditis elegans OX=6239 GN=mlc-1 PE=4 SV=1	1.3	0.5	34.1	5
MDHM_CAEEL	Probable malate dehydrogenase, mitochondrial OS=Caenorhabditis elegans OX=6239 GN=mdh-2 PE=3 SV=1	1.25	0.5	44.3	9
BCAT_CAEEL	Branched-chain-amino-acid aminotransferase, cytosolic OS=Caenorhabditis elegans OX=6239 GN=bcat-1 PE	1.23	0.5	36.1	12
EIF3M_CAEEL	COP9/Signalosome and eIF3 complex-shared subunit 1 OS=Caenorhabditis elegans OX=6239 GN=cif-1 PE=1	1.21	0.5	28.2	11
ARPC4_CAEEL	Probable actin-related protein 2/3 complex subunit 4 OS=Caenorhabditis elegans OX=6239 GN=arx-6 PE=3 S	1.19	0.5	23.1	5
SYNC_CAEEL	Asparagine--tRNA ligase, cytoplasmic OS=Caenorhabditis elegans OX=6239 GN=nars-1 PE=3 SV=1	1.18	0.5	31.7	15
F37C4_CAEEL	Protein F37C4.5 OS=Caenorhabditis elegans OX=6239 GN=F37C4.5 PE=1 SV=3	1.18	0.5	38.5	13
GUAA_CAEEL	Probable GMP synthase [glutamine-hydrolyzing] OS=Caenorhabditis elegans OX=6239 GN=gmps-1 PE=3 SV	1.17	0.5	35.2	19
TCTP_CAEEL	Translationally-controlled tumor protein homolog OS=Caenorhabditis elegans OX=6239 GN=tct-1 PE=1 SV=	1.13	0.5	38.1	5
SYK_CAEEL	Lysine--tRNA ligase OS=Caenorhabditis elegans OX=6239 GN=kars-1 PE=3 SV=1	1.09	0.5	28.8	13
HSP10_CAEEL	Heat shock protein Hsp-12.2 OS=Caenorhabditis elegans OX=6239 GN=hsp-12.2 PE=3 SV=1	1.09	0.5	28.2	3
6PGL_CAEEL	Putative 6-phosphogluconolactonase OS=Caenorhabditis elegans OX=6239 GN=Y57G11C.3 PE=3 SV=2	1.06	0.4	40.1	7
TERA2_CAEEL	Transitional endoplasmic reticulum ATPase homolog 2 OS=Caenorhabditis elegans OX=6239 GN=cdc-48.2 P	1.04	0.4	42.2	18
IMDH_CAEEL	Inosine-5~-monophosphate dehydrogenase OS=Caenorhabditis elegans OX=6239 GN=T22D1.3 PE=3 SV=2	1.04	0.4	29.2	12
ODP2_CAEEL	Dihydrolipoyllysine-residue acetyltransferase component of pyruvate dehydrogenase complex, mitochondrial (	1.04	0.4	29.4	11
LDH_CAEEL	L-lactate dehydrogenase OS=Caenorhabditis elegans OX=6239 GN=ldh-1 PE=2 SV=1	1.01	0.4	33.3	8
MYO1_CAEEL	Myosin-1 OS=Caenorhabditis elegans OX=6239 GN=myo-1 PE=1 SV=3	1	0.4	32.2	47
G3P3_CAEEL	Glyceraldehyde-3-phosphate dehydrogenase 3 OS=Caenorhabditis elegans OX=6239 GN=gpd-3 PE=3 SV=1	1	0.4	34.3	8
TERA1_CAEEL	Transitional endoplasmic reticulum ATPase homolog 1 OS=Caenorhabditis elegans OX=6239 GN=cdc-48.1 P	0.97	0.4	42.3	18
CATA2_CAEEL	Catalase-2 OS=Caenorhabditis elegans OX=6239 GN=ctl-1 PE=1 SV=3	0.95	0.4	37	12
ATPA_CAEEL	ATP synthase subunit alpha, mitochondrial OS=Caenorhabditis elegans OX=6239 GN=atp-1 PE=1 SV=1	0.94	0.4	20.6	11
MYO2_CAEEL	Myosin-2 OS=Caenorhabditis elegans OX=6239 GN=myo-2 PE=1 SV=2	0.92	0.4	36.2	43
SUR5_CAEEL	Acetoacetyl-CoA synthetase OS=Caenorhabditis elegans OX=6239 GN=sur-5 PE=2 SV=1	0.92	0.4	28.9	14
P4HA1_CAEEL	Prolyl 4-hydroxylase subunit alpha-1 OS=Caenorhabditis elegans OX=6239 GN=dpy-18 PE=1 SV=2	0.91	0.4	38.8	13
ODPA_CAEEL	Probable pyruvate dehydrogenase E1 component subunit alpha, mitochondrial OS=Caenorhabditis elegans O	0.91	0.4	40.6	9
MTSS1_CAEEL	Single-stranded DNA-binding protein, mitochondrial OS=Caenorhabditis elegans OX=6239 GN=mtss-1 PE=1	0.91	0.4	32.4	4
ALH13_CAEEL	Probable delta-1-pyrroline-5-carboxylate synthase OS=Caenorhabditis elegans OX=6239 GN=alh-13 PE=3 SV	0.87	0.4	23.9	16
NMT_CAEEL	Probable glycylpeptide N-tetradecanoyltransferase OS=Caenorhabditis elegans OX=6239 GN=nmt-1 PE=3 SV	0.87	0.4	37.6	10
CATZ1_CAEEL	Cathepsin Z-1 OS=Caenorhabditis elegans OX=6239 GN=cpz-1 PE=1 SV=1	0.87	0.4	27.5	7
EIF3G_CAEEL	Eukaryotic translation initiation factor 3 subunit G OS=Caenorhabditis elegans OX=6239 GN=eif-3.G PE=3 S	0.87	0.4	27.9	6
IDH3B_CAEEL	Probable isocitrate dehydrogenase [NAD] subunit beta, mitochondrial OS=Caenorhabditis elegans OX=6239 (	0.84	0.4	32.2	8
DLDH_CAEEL	Dihydrolipoyl dehydrogenase, mitochondrial OS=Caenorhabditis elegans OX=6239 GN=dld-1 PE=3 SV=2	0.83	0.3	26.3	9
HSP90_CAEEL	Heat shock protein 90 OS=Caenorhabditis elegans OX=6239 GN=daf-21 PE=1 SV=1	0.82	0.3	37.7	15
SUCB1_CAEEL	Succinate--CoA ligase [ADP-forming] subunit beta, mitochondrial OS=Caenorhabditis elegans OX=6239 GN=	0.82	0.3	35.9	9
TCPZ_CAEEL	T-complex protein 1 subunit zeta OS=Caenorhabditis elegans OX=6239 GN=cct-6 PE=1 SV=1	0.81	0.3	26.2	10
FABP6_CAEEL	Fatty acid-binding protein homolog 6 OS=Caenorhabditis elegans OX=6239 GN=lbp-6 PE=1 SV=1	0.8	0.3	36.3	3
ODBA_CAEEL	2-oxoisovalerate dehydrogenase subunit alpha, mitochondrial OS=Caenorhabditis elegans OX=6239 GN=bcl	0.79	0.3	38.7	9
VATA_CAEEL	V-type proton ATPase catalytic subunit A OS=Caenorhabditis elegans OX=6239 GN=vha-13 PE=1 SV=3	0.78	0.3	31.5	12
TCPG_CAEEL	T-complex protein 1 subunit gamma OS=Caenorhabditis elegans OX=6239 GN=cct-3 PE=3 SV=3	0.78	0.3	31.1	11
HSP7C_CAEEL	Heat shock 70 kDa protein C OS=Caenorhabditis elegans OX=6239 GN=hsp-3 PE=1 SV=2	0.77	0.3	28.4	13

ARP2_CAEEL	Actin-related protein 2 OS=Caenorhabditis elegans OX=6239 GN=arx-2 PE=3 SV=1	0.76	0.3	35.2	8
CLH_CAEEL	Probable clathrin heavy chain 1 OS=Caenorhabditis elegans OX=6239 GN=chc-1 PE=1 SV=1	0.74	0.3	27.4	33
SYQ_CAEEL	Probable glutamine--tRNA ligase OS=Caenorhabditis elegans OX=6239 GN=qars-1 PE=3 SV=1	0.72	0.3	28.6	15
VATH2_CAEEL	Probable V-type proton ATPase subunit H 2 OS=Caenorhabditis elegans OX=6239 GN=vha-15 PE=3 SV=1	0.7	0.3	37.4	9
FBRL_CAEEL	rRNA 2~-O-methyltransferase fibrillarin OS=Caenorhabditis elegans OX=6239 GN=fib-1 PE=2 SV=1	0.68	0.3	29	6
DYN1_CAEEL	Dynamin OS=Caenorhabditis elegans OX=6239 GN=dyn-1 PE=1 SV=3	0.67	0.3	20.5	13
SODM1_CAEEL	Superoxide dismutase [Mn] 1, mitochondrial OS=Caenorhabditis elegans OX=6239 GN=sod-2 PE=1 SV=1	0.66	0.3	24.9	3
PYC1_CAEEL	Pyruvate carboxylase 1 OS=Caenorhabditis elegans OX=6239 GN=pyc-1 PE=1 SV=1	0.64	0.3	27.2	19
ODPB_CAEEL	Pyruvate dehydrogenase E1 component subunit beta, mitochondrial OS=Caenorhabditis elegans OX=6239 GI	0.64	0.3	26.1	5
UGDH_CAEEL	UDP-glucose 6-dehydrogenase OS=Caenorhabditis elegans OX=6239 GN=sqv-4 PE=1 SV=1	0.62	0.3	25.2	7
PRS10_CAEEL	Probable 26S proteasome regulatory subunit 10B OS=Caenorhabditis elegans OX=6239 GN=rpt-4 PE=1 SV=:	0.62	0.3	31.3	7
VINC_CAEEL	Vinculin OS=Caenorhabditis elegans OX=6239 GN=deb-1 PE=1 SV=2	0.61	0.3	32.8	16
UBP7_CAEEL	Ubiquitin carboxyl-terminal hydrolase 7 OS=Caenorhabditis elegans OX=6239 GN=math-33 PE=3 SV=1	0.59	0.2	22.2	18
SYV_CAEEL	Valine--tRNA ligase OS=Caenorhabditis elegans OX=6239 GN=glp-4 PE=1 SV=1	0.58	0.2	31.1	15
ACLY_CAEEL	Probable ATP-citrate synthase OS=Caenorhabditis elegans OX=6239 GN=acly-1 PE=3 SV=1	0.57	0.2	26	17
LMN1_CAEEL	Lamin-1 OS=Caenorhabditis elegans OX=6239 GN=lmn-1 PE=1 SV=2	0.57	0.2	24.7	9
TBB4_CAEEL	Tubulin beta-4 chain OS=Caenorhabditis elegans OX=6239 GN=tbb-4 PE=3 SV=1	0.56	0.2	23.4	7
DIM_CAEEL	Disorganized muscle protein 1 OS=Caenorhabditis elegans OX=6239 GN=dim-1 PE=1 SV=3	0.56	0.2	20.8	10
ATPB_CAEEL	ATP synthase subunit beta, mitochondrial OS=Caenorhabditis elegans OX=6239 GN=atp-2 PE=1 SV=2	0.56	0.2	30.5	8
TCPB_CAEEL	T-complex protein 1 subunit beta OS=Caenorhabditis elegans OX=6239 GN=cct-2 PE=1 SV=2	0.56	0.2	27.8	8
CATA1_CAEEL	Peroxisomal catalase 1 OS=Caenorhabditis elegans OX=6239 GN=ctl-2 PE=1 SV=3	0.56	0.2	35	8
TCPD_CAEEL	T-complex protein 1 subunit delta OS=Caenorhabditis elegans OX=6239 GN=cct-4 PE=2 SV=1	0.55	0.2	29.8	7
CAND1_CAEEL	Cullin-associated NEDD8-dissociated protein 1 OS=Caenorhabditis elegans OX=6239 GN=cand-1 PE=1 SV=	0.54	0.2	24.3	19
TCPA_CAEEL	T-complex protein 1 subunit alpha OS=Caenorhabditis elegans OX=6239 GN=cct-1 PE=3 SV=2	0.54	0.2	21.9	8
CISY_CAEEL	Probable citrate synthase, mitochondrial OS=Caenorhabditis elegans OX=6239 GN=cts-1 PE=3 SV=1	0.54	0.2	26.3	7
PH4H_CAEEL	Phenylalanine-4-hydroxylase OS=Caenorhabditis elegans OX=6239 GN=pah-1 PE=1 SV=2	0.53	0.2	23	7
NOP56_CAEEL	Nucleolar protein 56 OS=Caenorhabditis elegans OX=6239 GN=nol-56 PE=3 SV=1	0.51	0.2	31.9	7
EIF3L_CAEEL	Eukaryotic translation initiation factor 3 subunit L OS=Caenorhabditis elegans OX=6239 GN=eif-3.L PE=3 SV	0.5	0.2	23.8	8
ECHM_CAEEL	Probable enoyl-CoA hydratase, mitochondrial OS=Caenorhabditis elegans OX=6239 GN=ech-6 PE=3 SV=1	0.5	0.2	23.6	4
SYIC_CAEEL	Isoleucine--tRNA ligase, cytoplasmic OS=Caenorhabditis elegans OX=6239 GN=iars-1 PE=3 SV=1	0.49	0.2	20.2	16
PCCA_CAEEL	Propionyl-CoA carboxylase alpha chain, mitochondrial OS=Caenorhabditis elegans OX=6239 GN=pcca-1 PE:	0.49	0.2	23.8	10
PDIA6_CAEEL	Protein disulfide-isomerase A6 homolog OS=Caenorhabditis elegans OX=6239 GN=pdi-6 PE=3 SV=1	0.49	0.2	29.1	6
ANM1_CAEEL	Protein arginine N-methyltransferase 1 OS=Caenorhabditis elegans OX=6239 GN=prmt-1 PE=1 SV=1	0.49	0.2	32.8	5
MTAP_CAEEL	S-methyl-5~-thioadenosine phosphorylase OS=Caenorhabditis elegans OX=6239 GN=B0228.7 PE=3 SV=2	0.49	0.2	20.8	4
MCCB_CAEEL	Probable methylcrotonoyl-CoA carboxylase beta chain, mitochondrial OS=Caenorhabditis elegans OX=6239 (	0.47	0.2	22.5	8
IF4E1_CAEEL	Eukaryotic translation initiation factor 4E-1 OS=Caenorhabditis elegans OX=6239 GN=ife-1 PE=1 SV=2	0.47	0.2	14.6	3
EIF3F_CAEEL	Eukaryotic translation initiation factor 3 subunit F OS=Caenorhabditis elegans OX=6239 GN=eif-3.F PE=3 SV	0.46	0.2	22.4	3
EIF3E_CAEEL	Eukaryotic translation initiation factor 3 subunit E OS=Caenorhabditis elegans OX=6239 GN=eif-3.E PE=3 S\	0.46	0.2	22.2	6
ACD11_CAEEL	Acyl-CoA dehydrogenase family member 11 OS=Caenorhabditis elegans OX=6239 GN=acdh-11 PE=1 SV=1	0.45	0.2	28.4	8
EGL4_CAEEL	cGMP-dependent protein kinase egl-4 OS=Caenorhabditis elegans OX=6239 GN=egl-4 PE=1 SV=2	0.45	0.2	22.8	10
GYS_CAEEL	Glycogen [starch] synthase OS=Caenorhabditis elegans OX=6239 GN=gsy-1 PE=1 SV=1	0.45	0.2	15.9	8
UCR1_CAEEL	Cytochrome b-c1 complex subunit 1, mitochondrial OS=Caenorhabditis elegans OX=6239 GN=ucr-1 PE=3 S\	0.45	0.2	17.2	6
RLA0_CAEEL	60S acidic ribosomal protein P0 OS=Caenorhabditis elegans OX=6239 GN=rla-0 PE=1 SV=3	0.45	0.2	24.4	4
P4HA2_CAEEL	Prolyl 4-hydroxylase subunit alpha-2 OS=Caenorhabditis elegans OX=6239 GN=phy-2 PE=1 SV=1	0.44	0.2	22.8	7
WDR4_CAEEL	tRNA (guanine-N(7)-)-methyltransferase non-catalytic subunit OS=Caenorhabditis elegans OX=6239 GN=wdr	0.44	0.2	18.6	5
GPDH2_CAEEL	Probable glycerol-3-phosphate dehydrogenase 2 OS=Caenorhabditis elegans OX=6239 GN=gpdh-2 PE=3 SV	0.44	0.2	29.6	5
SYRC_CAEEL	Probable arginine--tRNA ligase, cytoplasmic OS=Caenorhabditis elegans OX=6239 GN=rars-1 PE=3 SV=2	0.43	0.2	19.4	9
KINH_CAEEL	Kinesin heavy chain OS=Caenorhabditis elegans OX=6239 GN=unc-116 PE=2 SV=2	0.42	0.2	25.6	9
EIF3B_CAEEL	Eukaryotic translation initiation factor 3 subunit B OS=Caenorhabditis elegans OX=6239 GN=eif-3.B PE=3 S\	0.41	0.2	26.8	8
T23O_CAEEL	Tryptophan 2,3-dioxygenase OS=Caenorhabditis elegans OX=6239 GN=tdo-2 PE=1 SV=1	0.41	0.2	16.4	5
EIF3K_CAEEL	Eukaryotic translation initiation factor 3 subunit K OS=Caenorhabditis elegans OX=6239 GN=eif-3.K PE=2 S\	0.41	0.2	21.7	3
14332_CAEEL	14-3-3-like protein 2 OS=Caenorhabditis elegans OX=6239 GN=ftt-2 PE=1 SV=1	0.4	0.2	19	3
ER442_CAEEL	Endoplasmic reticulum resident protein 44.2 OS=Caenorhabditis elegans OX=6239 GN=erp-44.2 PE=1 SV=2	0.4	0.2	25.2	5
IDH3A_CAEEL	Probable isocitrate dehydrogenase [NAD] subunit alpha, mitochondrial OS=Caenorhabditis elegans OX=6239	0.39	0.2	19.3	4
CYP3_CAEEL	Peptidyl-prolyl cis-trans isomerase 3 OS=Caenorhabditis elegans OX=6239 GN=cyn-3 PE=1 SV=1	0.39	0.2	11	2
FAD1_CAEEL	Probable FAD synthase OS=Caenorhabditis elegans OX=6239 GN=flad-1 PE=3 SV=1	0.38	0.2	24.5	6
CGH1_CAEEL	ATP-dependent RNA helicase cgh-1 OS=Caenorhabditis elegans OX=6239 GN=cgh-1 PE=1 SV=1	0.38	0.2	25.6	5
ALF1_CAEEL	Fructose-bisphosphate aldolase 1 OS=Caenorhabditis elegans OX=6239 GN=aldo-1 PE=1 SV=1	0.38	0.2	13.7	4
EIF3A_CAEEL	Eukaryotic translation initiation factor 3 subunit A OS=Caenorhabditis elegans OX=6239 GN=egl-45 PE=3 SV	0.36	0.2	25.2	12
EIF3C_CAEEL	Eukaryotic translation initiation factor 3 subunit C OS=Caenorhabditis elegans OX=6239 GN=eif-3.C PE=3 S\	0.36	0.2	17.4	9
ZPR1_CAEEL	Zinc finger protein ZPR1 homolog OS=Caenorhabditis elegans OX=6239 GN=W03F9.1 PE=3 SV=2	0.36	0.2	18.2	5

***** MASCOT protein identification and molar fraction quantification performed for on a 50 µL sample of standard reaction mixture (50 µM acetyl-CoA, 50 µM ATP, 50 mM NH_4HCO_3, 10 mM $MgCl_2$), after addition of 10 µL of *C. elegans* protein extract (protein content 2.9 µg/µL) and subsequent reaction. The highlighted rows indicate enzymes that can be responsible for acetyl-CoA depletion.

Besides ACC (acetyl-CoA carboxylase, from *C. elegans pod-2* gene), the list reports acetyl-CoA acetyltransferase (from *C. elegans kat-1* gene) and acetoacetyl-CoA synthetase (from *C. elegans sur-5* gene). Below the quantification threshold, also acetyl-CoA synthetase (from *C. elegans acs-19* gene) and acetyl-CoA hydrolase (from *C. elegans acer-1* gene) were identified. However only acetyl-CoA acetyltransferase and acetyl-CoA hydrolase are likely to contribute to the experimentally observed consumption of acetyl-CoA (see main text), because acetoacetyl-CoA synthetase, which catalyzes the decarboxylative condensation of acetyl-CoA and malonyl-CoA (the first reaction of fatty acid anabolism), would need malonyl-CoA as additional substrate, whereas acetyl-CoA synthetase would need AMP (adenosine monophosphate) and pyrophosphate to split acetyl-CoA into acetate and free CoA and to reform ATP.

Heading abbreviations: prot_acc = Protein account; prot_desc = Protein decription; amPAI = number of peptides identified divided by one tenth the number of theoretically observable tryptic peptides; %mol-Prot = Protein molar percentage; prot_cover % = Protein sequence coverage; prot_sequences_sig = Number of identified peptides.

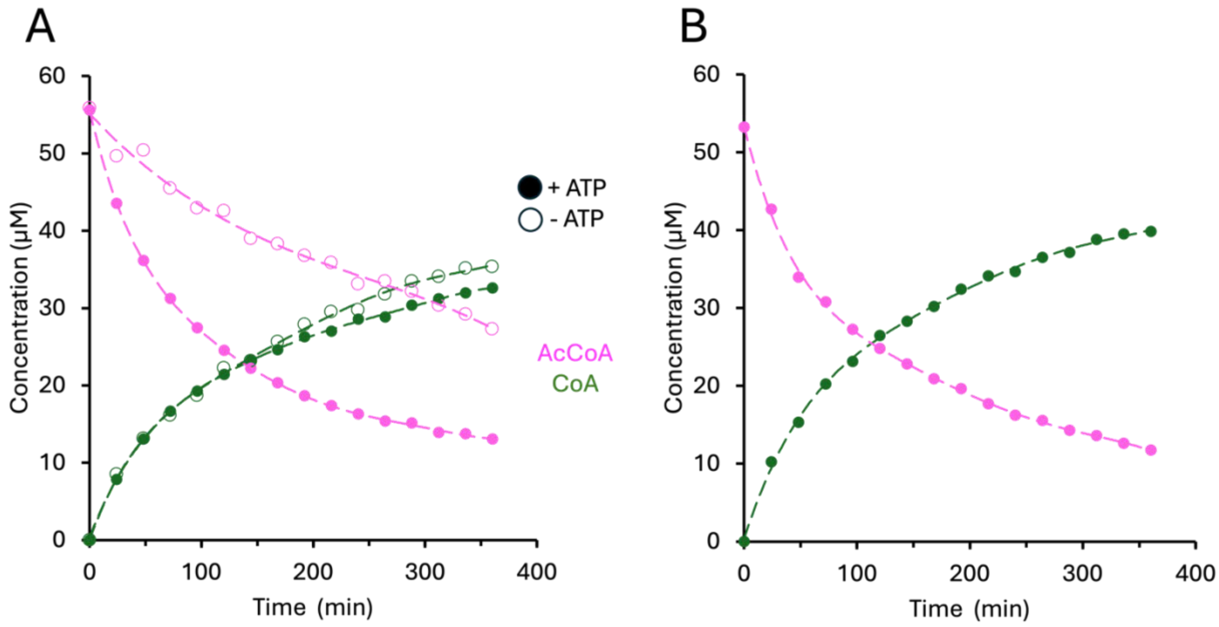

Figure S1. ACC inhibition and concentration progress curves. A) HPLC quantification of acetyl-CoA depletion and CoA formation in the presence (filled symbols) and absence (empty symbols) of ATP. B) HPLC quantification of acetyl-CoA depletion and CoA formation under conditions of ACC inhibition with 50 µM avocadene acetate. The dashed lines are polynomials drawn as eye guides.

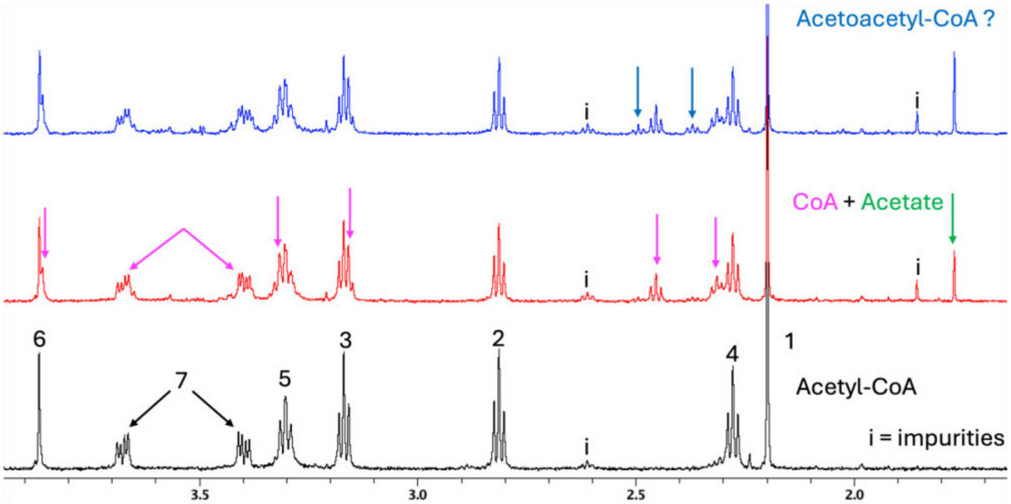

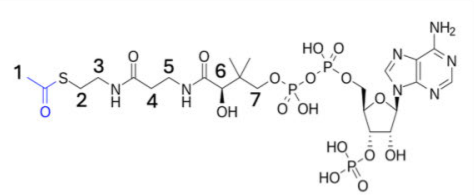

Figure S2. ACC-independent acetyl-CoA depletion. Aliphatic region from ^{1}H NMR spectra of 50 µM acetyl-CoA without (black trace) and with addition of different batches of C. elegans protein extract (red and blue traces). The individual assignments are reported only for the spectrum recorded without the protein extract (but with 30 mM NH_4HCO_3). The additional species that are formed after about 18 hours from the addition of the protein extracts are indicated by arrows.

Note S1. ACAT and ACH activity. Figure S2 shows diagnostic features of the NMR spectra obtained from reaction mixtures before or after the addition of the *C.elegans* protein extract containing ACC. No ATP was added to make sure that acetyl-CoA depletion was only attributable to ATP-independent sources, which should primarily exclude substrate consumption due to ACC. With different preparations of *C. elegans* protein extract some deviations were appreciated because of the different composition of the extract batches (e.g. Table S2). ACH (acetyl-CoA hydrolase) always appeared responsible for the largest amount of acetyl-CoA consumption measured in the absence of ATP. This conclusion could be drawn from spectra such as those shown above, where the red middle trace shows the new resolved and unresolved signals (marked by arrows) observed after addition of the *C. elegans* protein extract. The detection of new signals, including the clearly resolved ones from the CH_2 in position 2 and 4 of a CoA moiety and the free acetate methyl one at ~ 1.8 ppm, was simultaneous and parallel to the decrease of the acetyl-CoA methyl peak at ~ 2.2 ppm, strongly suggesting that the whole pattern can be attributed to the ACH-catalyzed hydrolysis of acetyl-CoA thioester into free CoA and acetate. On the other hand, the top blue trace displays the accumulation of a further species exhibiting two additional resolved signals, again likely to arise from positions 2 and 4 of a CoA moiety possibly belonging to the acetoacetyl-CoA produced by ACAT (acetyl-CoA acetyl transferase) catalysis. These signals appear less intense compared to the corresponding ones of free CoA that are visible in the same trace. Notably, the stoichiometry of the ACAT-catalyzed reaction entails the consumption of two acetyl-CoA molecules, whereas only one molecule is consumed in the ACH-catalyzed reaction. However, no quantitative conclusion on the relative, individual contribution of ACAT and ACH to substrate depletion could be safely reached from these data or from data similar to those illustrated in Fig. S1. On the contrary, the data illustrated in panels A and B of Fig. 3 and Fig. 5 of main text allow assessing the ACC contribution to the substrate depletion. In absolute terms, at 25ºC, based on the HPLC estimates, ACC consumes ~ 35% (without inhibitor) or ~ 14% (with 50 µM inhibitor) of the total acetyl-CoA depletion, amounting on average to 41.6±0.4 µM, in the absence and presence of inhibitor. At 20ºC, based on the NMR estimates, ACC consumes ~ 25% (without inhibitor) or ~ 10% (with 25 µM inhibitor) of the total acetyl-CoA depletion (39.5±1.0 µM, on average, in the absence and presence of inhibitor). The total substrate consumption was essentially independent of the inhibitor presence for the HPLC-monitored and the NMR-monitored experiments. Over intervals of 16-18 hours, in fact, the substrate depletion of the NMR samples with halved inhibitor concentration with respect to

substrate reached the same plateau as in the absence of inhibitor. It is also worth noting the constant reduction factor (2.5) of the ACC contribution to substrate depletion in the presence of the inhibitor.

Table S3 – Concentration time-course fitting*

[Substrate] / [Inhibitor]	$[S_0]$ μM	K_M μM	V_{max} $\mu M \times min^{-1} \times mg^{-1}$	R^2
**HPLC-based Determinations**				
1:0				
	15.50	9.112	118	0.9980
	14.09	7.230	97.0	0.9980
	15.00	6.302	93.2	0.9981
AVERAGES	14.88±0.74	7.548±1.432	103±14	0.9980
1:1				
	6.798	1.916	95.1	0.9645
	6.424	0.948	70.5	0.9277
	5.659	1.782	74.9	0.9526
AVERAGES	6.293±0.581	1.549±0.525	80.2±13.2	0.9483
**NMR-based Determinations**				
1:0				
	7.637	4.172	22.8	0.9659
	9.523	5.545	22.5	0.9837
	9.562	4.711	25.0	0.9826
AVERAGES	8.907±1.100	4.809±0.692	23.5±1.4	0.9774
1:0.5				
	4.736	3.101	23.7	0.9459
	4.117	2.696	20.6	0.9459
	5.037	3.278	13.9	0.9830
AVERAGES	4.630±0.470	3.025±0.298	19.4±5.0	0.9583

***** The fitting was performed with the software Prism 10 (v. 10.2.3) (GraphPad Software LLC), according to equations S3 and S6 reported in Note S2. For each individual HPLC-based determination of the triplicate series, 16 experimental points were collected, corresponding to 13 degrees of freedom for the fitting. For the NMR-based determinations, 98-106 experimental points were collected, allowing for 95-103 degrees of freedom for the fitting. The listed V_{max} values are expressed as rate of product formation per mg of enzyme, after converting the fitting results obtained from samples with 29 µg protein content (65.3 µg for NMR samples) and 8.1% ACC mass percentage (2.5% for NMR samples). All the listed values are given as they appear in the fitting output. Only two significant figures should be safely allowed in the fitted parameter averages of each triplicate series, as reported in Table 1 of the main text. The standard deviations are reported as error on the averages.

Note S2. Fitting equation. Under conditions of steady-state approximation,[1] the rate (*v*) of an enzyme-catalyzed reaction is expressed in terms of the time (*t*) dependence of the substrate concentration ([*S*]) and the Michaelis-Menten parameters, K_M and V_{max} :

$$v = -\frac{d[S]}{dt} = V_{max}\frac{[S]}{[S] + K_M}$$

Eq. {S1}

whose integration

$$K_M \ln\frac{[S_0]}{[S]} + [S_0] - [S] = V_{max}t$$

Eq. {S2a}

represents an implicit solution where [S_0] is the initial (effective) substrate concentration. Recasting of the implicit solution as:

$$\frac{[S]}{K_M} exp\left(\frac{[S]}{K_M}\right) = \left(\frac{[S_0]}{K_M} exp\left(\frac{[S_0] - V_{max}t}{K_M}\right)\right)$$

Eq. {S2b}

enables the introduction of the Lambert *W* function[2] which allows one to obtain an explicit analytical solution for the substrate concentration, [*S*]:

$$[S] = K_M W(x) \qquad x = \left(\frac{[S_0]}{K_M} exp\left(\frac{[S_0] - V_{max}t}{K_M}\right)\right)$$

Eq. {S3}

where *W(x)= y* is defined as the inverse of the function satisfying:

$$ye^y = x \quad \text{i.e.} \quad W(x)e^{W(x)} = x$$

Eq. {S4}

in analogy to the inverse relationship between the exponential and the natural logarithm functions.
The substrate concentration can also be replaced by the product concentration according to:

$$[P] = [S_0] - [S]$$

Eq. {S5}.

W(x) can be approximated by analytical expressions,[3] and this enables direct fitting of experimental data. Among the different options,[3,4] the analytical approximation that was selected to fit the experimental data was:

$$W(x) \approx \ln(1 + x)\left\{1 - \frac{ln(1 + \ln(1 + x))}{2 + \ln(1 + x)}\right\}$$

Eq. {S6}

with x expressed as defined in Eq. {S3}.

This expression was employed to fit the HPLC- and NMR-derived time-progress curves of malonyl-CoA concentration in the absence and presence of inhibitor.

Besides the K_M and V_{max} values, also the initial substrate concentration, $[S_0]$, was included among the fitting targets because the simultaneous competition of other enzymatic activities for acetyl-CoA (see main text) decreases the actually available substrate concentration with respect to the total dissolved amount (~50 µM).

As expected, any attempt to fit the time-courses of acetyl-CoA or free CoA determined by HPLC (if the possible contribution to CoA intensity from acetoacetyl-CoA could be neglected), or of acetate and acetyl-CoA based on NMR data, with the same approach failed, consistently with the composite nature of the corresponding formation or transformation processes. The possibility of using the independently determined CoA and acetate concentration time-courses to analyze the additional processes (with respect to the ACC activity) that use the acetyl-CoA substrate was not considered because of the different conditions of the HPLC and NMR determinations (enzyme contents of the extracts and temperature).

Table S4. ***C. elegans*** **ACC kinetic parameters from malonyl-CoA concentration time progress compared with previous determinations***

T ºC	K_M μM	V_{max} $\mu M \times min^{-1} \times mg^{-1}$	Organism / Enzyme type Inhibitor / Monitoring	Reference
25	7.5±1.4	(1.0±0.1) ×10^2	*C. elegans* / L4 extract ACC/ No Inhibitor / HPLC	This work
25	1.5±0.5	80±13	*C. elegans* / L4 extract ACC / + Avocadene Acetate / HPLC	This work
20	4.8±0.7	23±1	*C. elegans* / L4 extract ACC/ No Inhibitor / NMR	This work
20	3.0±0.3	19±5	*C. elegans* / L4 extract ACC/ + Avocadene Acetate / NMR	This work
			REPORTED DETERMINATIONS Organism / Enzyme type	
37	4.0	-	Rat / Purified Liver ACC	(5)
37	31.7±1.5	-	Rat / Purified Skeletal muscle ACC	(6)
37	21.5±1.0	-	Rat / Purified Adipose Tissue ACC	(6)
37	34±4	-	Human / Recombinant ACC1	(7)
37	58±17	-	Human / Recombinant ACC2	(7)
37	37±12	-	Rat / Recombinant ACC2	(7)
25	2.0±0.2	-	Human / Recombinant ACC2 [27-2458]	(8)
25	2.6±0.8	-	Human / Recombinant ACC2 [21-2458]	(8)
25	0.85±0.27	-	Chicken / Purified Liver ACC**	(9)
30	97.9±11.8	0.489±0.0162	*Streptomyces coelicolor* / Purified and reconstituted Acyl-CoA Carboxylase	(10)
37	1.5±0.2	173±12	*Thermobifida fusca YX* / Recombinant Acyl-CoA Carboxylase	(11)

*All reported K_M values refer to acetyl-CoA substrate. In only two cases were the K_M values measured in the presence of inhibitors (avocadene acetate for *C. elegans* ACC and malonyl-CoA for chicken liver ACC). As explicitly indicated by the units, the V_{max} values are given per mg of enzyme. For the HPLC-based estimates, the V_{max} values obtained from the fitting were scaled to 1 mg of enzyme based on the overall protein content (2.9 μg/μL) and the ACC mass percentage (8.1%) of the experimental protein extract samples. For the NMR-based estimates, the same procedure was adopted using the proper protein content of the employed extract (1.72 μg/μL) and assuming a value of 2.5% for the ACC mass percentage, as obtained from the initial slopes of the concentration evolution (see text). The listed kinetic parameters are averages ± standard deviations, obtained from the fitting of data collected in triplicate at substrate/inhibitor ratio 1:0 and 1:1 (HPLC) or 1:0 and 1:0.5 (NMR).

** In the presence of malonyl-CoA.

REFERENCES

1. Briggs, G. E.; Haldane, J. B. S., A Note on the Kinetics of Enzyme Action. *Biochem J.* **1925**, *19*, 338-339.

2. Corless, R. M; Gonnet, G. H.; Hare, D. E. G.; Jeffry, D. J.; Knuth, D. E. On the Lambert W function. *Adv. Comput. Math.* **1996**, *5*, 329-359.

3. Winitzki, S. Uniform approximations for transcendental functions. *Lecture Notes Comp. Sci.* **2003**, *2667*, 780-789.

4. Golicnik, M. Explicit reformulations of time-dependent solutions for a Michaelis-Menten enzyme reaction model. *Anal. Biochem.* **2010**, *406*, 94-96.

5. Yeh L. A.; Kim, K-H. Regulation of Acetyl-CoA Carboxylase: Properties of CoA Activation of Acetyl-CoA Carboxylase. *Proc. Natl. Acad. Sci. USA* **1980**, *77*, 3351-3355.

6. Trumble, G. E.; Smith, M. A.; Winder, W. W. Purification and characterization of rat skeletal muscleacetyl-CoA carboxylase. *Eur. J. Biochem.* **1995**, *231*, 192-198.

7. Cheng, D.; Chu, C. H.; Chen, L.; Feder, J. N.; Mintier, G. A.; Wu, Y.; Cook, J. W.; Harpel, M. R.; Locke, G. A.; An, Y.; Tamura, J. K. Expression, purification and characterization of human and rat acetyl coenzyme A carboxylase (ACC) isozymes. *Protein Expr. Purif.* **2007**, *51*, 11-21.

8. Kim, K. W.; Yamane, H.; Zondlo, J.; Busby, J.; Wang, M. Expression, purification and characterization of human acetyl-CoA carboxylase 2. *Protein Expr. Purif.* **2007**, *53*, 16-23.

9. Cox, B. G.; Hammes, G. G. Steady-state kinetic study of fatty acid synthase. *Proc. Natl. Acad. Sci. USA* **1983**, *80*, 4233-4237.

10. Diacovich, L.; Peirù, S.; Kurth, D.; Rogriguez, E.; Podestà, F.; Koshla, C.; Gramajo, H. Kinetic and Structural Analysis of a New Group of Acyl-CoA Carboxylases Found in *Streptomyces coelicolor* A3(2). *J. Biol. Chem.* **2002**, *277*, 31228-31236.

11. Shivaiah, K-K.; Upton, B.; Nikolau, B. J. Kinetic, Structural, and Mutational Analysis of Acyl-CoA Carboxylase From *Thermobifida fusca* YX. *Front. Mol. Biosci.* **2021**, *7*, 615614.
